# Supplementary material for: A Proof-of-Concept for Epigenetic Therapy of Tissue Fibrosis: Inhibition of Liver Fibrosis Progression by 3-Deazaneplanocin A
Source: Mol Ther. 2017 Jan 4;25(1):218–31. doi: 10.1016/j.ymthe.2016.10.004 (PMC5363305; doi:10.1016/j.ymthe.2016.10.004)
Supplement: Document S1. Figures S1–S7 and Tables S1 and S2 [file mmc1.pdf]

YMTHE, Volume *25*

Supplemental Information

A Proof-of-Concept for Epigenetic Therapy of Tissue Fibrosis: Inhibition of Liver Fibrosis Progression by 3-Deazaneplanocin A

Müjdat Zeybel, Saimir Luli, Laura Sabater, Timothy Hardy, Fiona Oakley, Jack Leslie, Agata Page, Eva Moran Salvador, Victoria Sharkey, Hidekazu Tsukamoto, David C.K. Chu, Uma Sharan Singh, Mirco Ponzoni, Patrizia Perri, Daniela Di Paolo, Edgar J. Mendivil, Jelena Mann, and Derek A. Mann

Supplementary material for manuscript "**A Proof-of-Concept for Epigenetic Therapy of Chronic Liver Disease: Inhibition of Fibrosis Progression by 3-Deazanoplanocin A (DZNep)**"

**Compound. No.**	**Structure**
**1.**	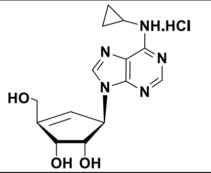
**2.**	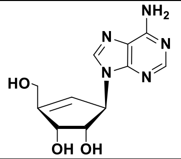
**3.**	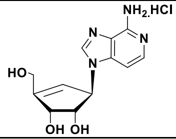
**4.**	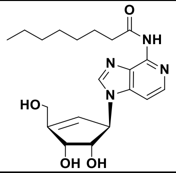
**5.**	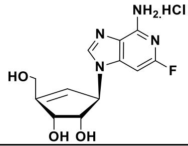
**6.**	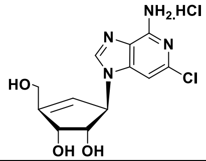
**7.**	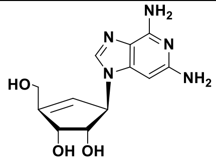
**8.**	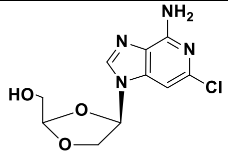
**9.**	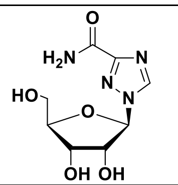

Supplementary Figure 1 – Chemical structures of compounds used in Figure 1.

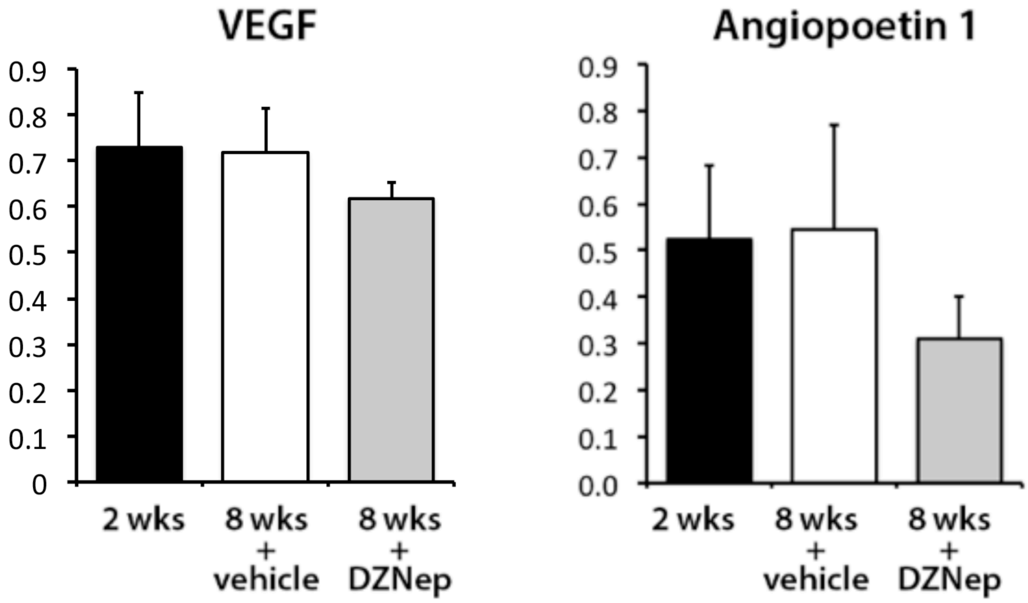

Supplementary Figure 2 –Angiopoietin I and VEGF expression in livers of mice treated with vehicle or DZNep in chronic CCl_4 model of liver fibrosis.

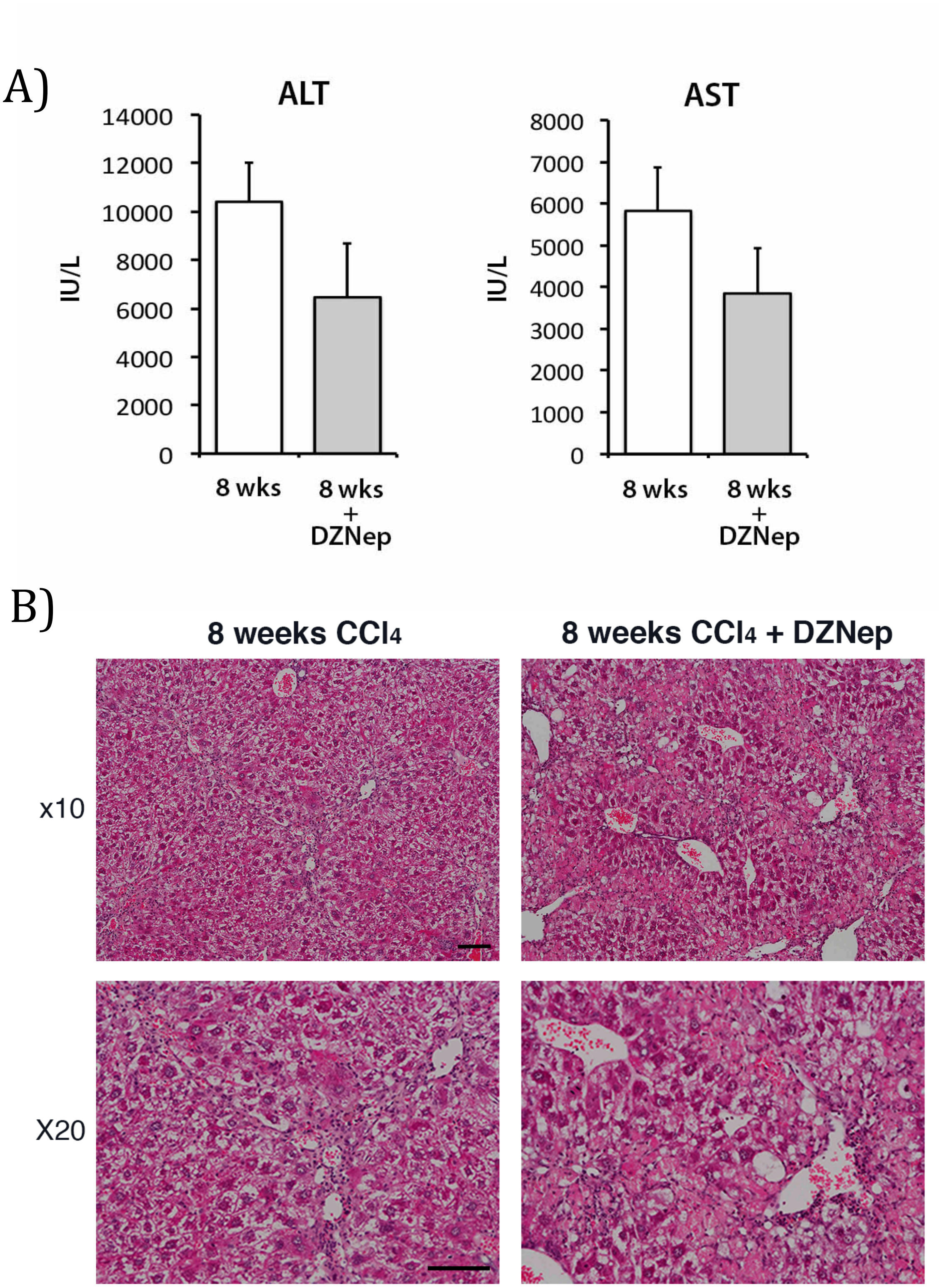

<u>Supplementary Figure 3</u> – A) ALT and AST values for mice treated with DZNep in chronic CCl_4 model of liver fibrosis. B) Representative Hematoxylin and Eosin stained slides of 8 week CCl4 injured livers, with or without DZNep (or vehicle)

in bottom panels. Amplification is 10X in top two pictures and 20X in the bottom ones. Scale bar – 100mm.

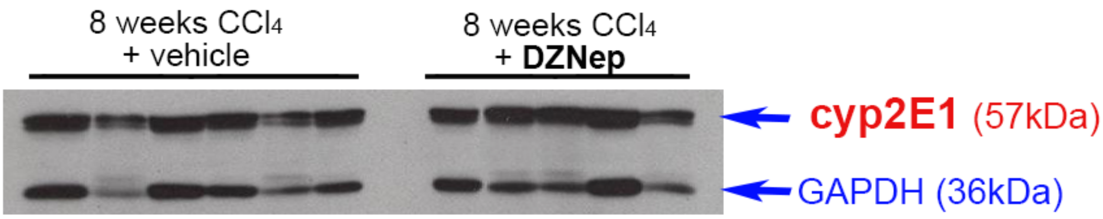

Supplementary Figure 4- Western blot for cyp2E1 expression in livers of animals receiving chronic CCl_4 with vehicle or DZNep treatment

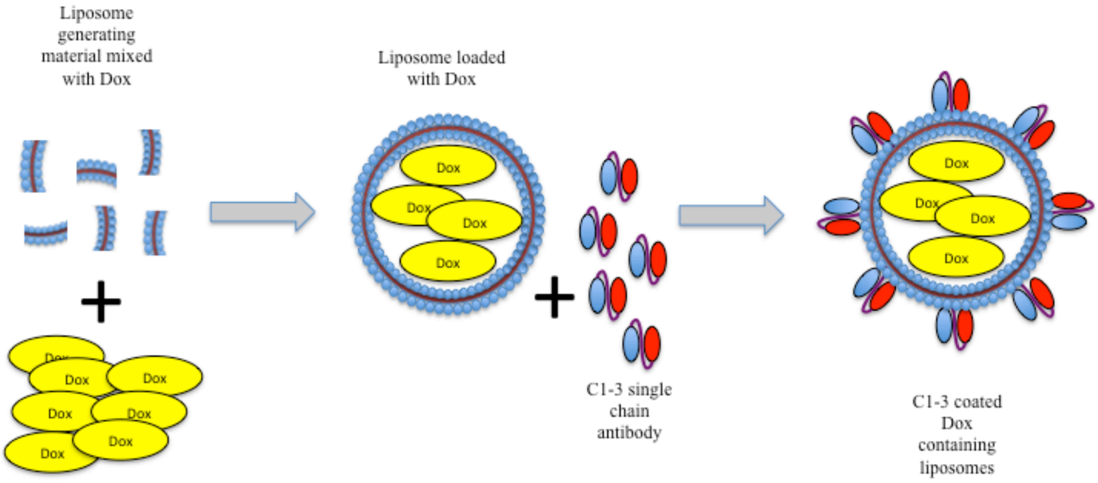

Supplementary Figure 5- Schematic of the C1-3 coated doxorubicin containing liposomes

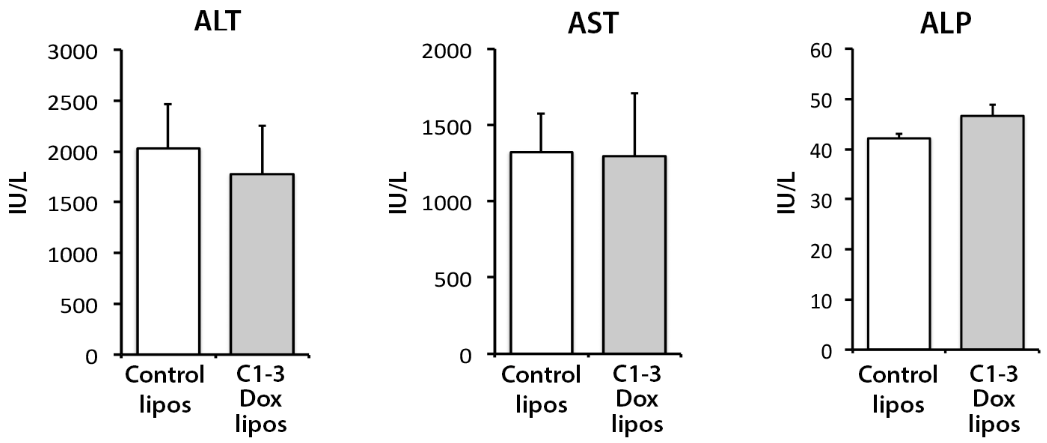

Supplementary Figure 6- ALT, AST and ALP values for mice treated with C1-3/empty liposomes (control liposomes) or C1-3/doxorubicine containing liposomes in acute CCl_4 liver injury

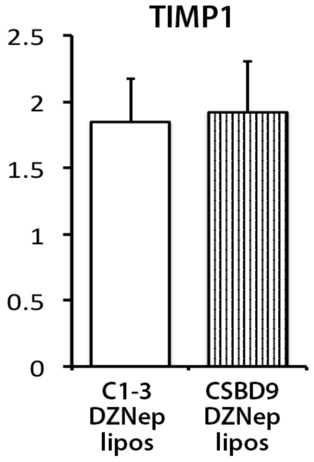

Supplementary Figure 7- TIMP1 expression in livers of mice treated with C1-3/DZNep liposomes or CSBD9/DZNep liposomes in chronic CCl_4 model of liver fibrosis

<u>**Table 2- Genes upregulated in DZNep treated livers (Sorted via fold change)**</u>

Gene name	Fold change
G6pc, glucose-6-phosphatase, catalytic, 3370255	4.4897
Hamp2, hepcidin antimicrobial peptide 2, 7330482	3.9385
Cyp4a14, cytochrome P450, family 4, subfamily a, polypeptide 14, 1940273	3.3069
Thrsp, thyroid hormone responsive SPOT14 homolog (Rattus), 6580403	3.2759
Cyp8b1, cytochrome P450, family 8, subfamily b, polypeptide 1, 4900341	3.1924
Cyp7a1, cytochrome P450, family 7, subfamily a, polypeptide 1, 4880333	3.1865
Cyp2c37, cytochrome P450, family 2. subfamily c, polypeptide 37, 4480437	3.0023
Cyp2c50, cytochrome P450, family 2, subfamily c, polypeptide 50, 290437	2.9735
Cyp2c37, cytochrome P450, family 2. subfamily c, polypeptide 37, 1240592	2.9628
Car3, carbonic anhydrase 3, 1450242	2.8145
2810007J24Rik, RIKEN cDNA 2810007J24 gene, 7610520	2.738
Acss2, acyl-CoA synthetase short-chain family member 2, 4570333	2.6234
Inmt, indolethylamine N-methyltransferase, 2360050	2.6051
Bhmt, betaine-homocysteine methyltransferase, 2480039	2.5697
Upp2, uridine phosphorylase 2, 2070372	2.53
Aqp8, aquaporin 8, 2470717	2.4786
Serpina6, serine (or cysteine) peptidase inhibitor, clade A, member 6, 6660403	2.4424
Cyp2c29, cytochrome P450, family 2, subfamily c, polypeptide 29, 4730403	2.3787
Upp2, uridine phosphorylase 2, 3520382	2.364
Slc25a25, solute carrier family 25 (mitochondrial carrier, phosphate carrier), member 25, 3440070	2.2409
Chrna4, cholinergic receptor, nicotinic, alpha polypeptide 4, 2260082	2.2151
Aqp8, aquaporin 8, 7160093	2.1861
Uhrf1, ubiquitin-like, containing PHD and RING finger domains, 1, 4560397	2.1823
Upp2, uridine phosphorylase 2, 1410170	2.0986
Raet1b, retinoic acid early transcript beta, 50025	2.0245
Cyp1a2, cytochrome P450, family 1, subfamily a, polypeptide 2, 5260367	1.9878
Slc2a2, solute carrier family 2 (facilitated glucose transporter), member 2, 2680593	1.9873
Cyp1a2, cytochrome P450, family 1, subfamily a, polypeptide 2, 1050079	1.9858
Raet1b, retinoic acid early transcript beta, 10541	1.9722
Gstm2, glutathione S-transferase, mu 2, 2690025	1.9349
Chrna4, cholinergic receptor, nicotinic, alpha polypeptide 4, 2070482	1.9326

Cyp4a31, cytochrome P450, family 4, subfamily a, polypeptide 31, 60364	1.8999
Gstm2, glutathione S-transferase, mu 2, 7510072	1.8703
Elovl6, ELOVL family member 6, elongation of long chain fatty acids (yeast), 670608	1.8645
Cyp4f14, cytochrome P450, family 4, subfamily f, polypeptide 14, 4640041	1.8626
Igfbp2, insulin-like growth factor binding protein 2, 580364	1.861
Aacs, acetoacetyl-CoA synthetase, 7650468	1.8587
Mcm6, minichromosome maintenance deficient 6 (MIS5 homolog, S. pombe) (S. cerevisiae), 3990243	1.8521
Upp2, uridine phosphorylase 2, 6040400	1.803
Mcm5, minichromosome maintenance deficient 5, cell division cycle 46 (S. cerevisiae), 6220270	1.7868
Rnf125, ring finger protein 125, 2320176	1.7865
Aldh1a1, aldehyde dehydrogenase family 1, subfamily A1, 5810470	1.7734
Gstm2, glutathione S-transferase, mu 2, 730025	1.7644
Gstt3, glutathione S-transferase, theta 3, 2350324	1.7562
Hpd, 4-hydroxyphenylpyruvic acid dioxygenase, 2360528	1.749
Pygl, liver glycogen phosphorylase, 1030142	1.7436
Agxt, alanine-glyoxylate aminotransferase, 6180408	1.7402
Psma3, proteasome (prosome, macropain) subunit, alpha type 3; 3' UTR, 5900047	1.7292
Rrm2, ribonucleotide reductase M2, 3440725	1.7222
Lss, lanosterol synthase, 5890553	1.7121
Sucnr1, succinate receptor 1, 1190148	1.7105
Mcm6, minichromosome maintenance deficient 6 (MIS5 homolog, S. pombe) (S. cerevisiae), 3290437	1.7096
Khk, ketohexokinase, 20010	1.7084
Khk, ketohexokinase, 6840014	1.7051
Psen2, presenilin 2, 990682	1.7015
Acly, ATP citrate lyase, 7050274	1.6998
Igfbp2, insulin-like growth factor binding protein 2, 1230240	1.6886
Fdps, farnesyl diphosphate synthetase, 5290671	1.6818
Dmgdh, dimethylglycine dehydrogenase precursor, 2450750	1.6815
Cyp2c70, cytochrome P450, family 2, subfamily c, polypeptide 70, 4280722	1.6791
Pah, phenylalanine hydroxylase, 1980328	1.6775
Ttc39c, tetratricopeptide repeat domain 39C, 7330228	1.6676
Slc47a1, solute carrier family 47, member 1, 6520022	1.6615
Scd1, stearoyl-Coenzyme A desaturase 1, 3890274	1.6552
Paqr9, progestin and adipoQ receptor family member IX, 650731	1.6489
Ces2g, carboxylesterase 2G, 7100458	1.6421
LOC100040592, PREDICTED: Mus musculus similar to Hmgcs1 protein, transcript variant 1 (LOC100040592), mRNA., 6940521	1.641

Paox, polyamine oxidase (exo-N4-amino), 3140102	1.6408
Insig1, insulin induced gene 1, 10309	1.6386
Gstt1, glutathione S-transferase, theta 1, 430564	1.6366
Dhcr7, 7-dehydrocholesterol reductase, 4280112	1.633
Nudt7, nudix (nucleoside diphosphate linked moiety X)-type motif 7, 5420685	1.6288
Tk1, thymidine kinase 1, 7400142	1.6261
Cyp2c67, cytochrome P450, family 2, subfamily c, polypeptide 67, 1170600	1.6232
Aldh1l1, aldehyde dehydrogenase 1 family, member L1, 380754	1.6209
Dhcr24, 24-dehydrocholesterol reductase, 4250228	1.6131
Idi1, Mus musculus isopentenyl-diphosphate delta isomerase (Idi1), transcript variant 2, mRNA., 3140768	1.6109
Slco1b2, solute carrier organic anion transporter family, member 1b2, 4570053	1.6097
Dak, dihydroxyacetone kinase 2 homolog (yeast), 1660739	1.6067
Bbox1, butyrobetaine (gamma), 2-oxoglutarate dioxygenase 1 (gamma-butyrobetaine hydroxylase), 450072	1.604
Slc22a1, solute carrier family 22 (organic cation transporter), member 1, 4250100	1.6039
Hsd17b6, hydroxysteroid (17-beta) dehydrogenase 6, 4060364	1.6017
LOC100044204, PREDICTED: Mus musculus hypothetical protein LOC100044204 (LOC100044204), mRNA., 3830048	1.5969
Mcm6, minichromosome maintenance deficient 6 (MIS5 homolog, S. pombe) (S. cerevisiae), 270379	1.5969
Pmvk, phosphomevalonate kinase, 670025	1.5963
Apol9b, apolipoprotein L 9b, 4280093	1.5951
Ugt1a6a, UDP glucuronosyltransferase 1 family, polypeptide A6A, 1170349	1.5777
Rdh11, retinol dehydrogenase 11, 650411	1.5716
Gulo, gulonolactone (L-) oxidase, 6650674	1.571
Idh2, isocitrate dehydrogenase 2 (NADP+), mitochondrial, 2510390	1.559
Hist1h2ad, histone cluster 1, H2ad, 3520717	1.5586
Hist1h2ap, histone cluster 1, H2ap, 6510253	1.5585
Ndrg2, N-myc downstream regulated gene 2, 1450601	1.5584
Adh4, alcohol dehydrogenase 4 (class II), pi polypeptide, 6840193	1.5574
Pygl, liver glycogen phosphorylase, 3310333	1.5572
Gsta3, glutathione S-transferase, alpha 3, 5550075	1.5566
Ddt, D-dopachrome tautomerase, 1990731	1.5565
Kynu, kynureninase (L-kynurenine hydrolase), 520138	1.5528
Cml1, camello-like 1, 7380671	1.5504
Mcm4, minichromosome maintenance deficient 4 homolog (S. cerevisiae), 2320368	1.5482
Abcb11, ATP-binding cassette, sub-family B (MDR/TAP), member 11,	1.5433

6330731	
Ces1g, carboxylesterase 1G, 6480397	1.5428
Dhcr24, NA, 2100162	1.5404
Hist1h2an, histone cluster 1, H2an, 4610129	1.5393
Acat2, acetyl-Coenzyme A acetyltransferase 2, 110661	1.5386
LOC100047200, PREDICTED: Mus musculus similar to T-box 3 protein (LOC100047200), mRNA., 6510162	1.538
Cyp3a25, cytochrome P450, family 3, subfamily a, polypeptide 25, 7570017	1.5379
Fignl1, fidgetin-like 1, 670500	1.5364
Dhdh, dihydrodiol dehydrogenase (dimeric), 3060066	1.536
Aox3, aldehyde oxidase 3, 3190646	1.5337
Cmbl, carboxymethylenebutenolidase-like (Pseudomonas), 5910072	1.5336
Cyp2b9, cytochrome P450, family 2, subfamily b, polypeptide 9, 1940504	1.5287
Ly6d, lymphocyte antigen 6 complex, locus D, 2510646	1.5262
Paqr9, progestin and adipoQ receptor family member IX, 3440739	1.5238
Hist1h2af, histone cluster 1, H2af, 4250711	1.5222
Tcf19, transcription factor 19, 6330725	1.5213
Nat8, N-acetyltransferase 8 (GCN5-related, putative), 3170255	1.5207
E2f1, E2F transcription factor 1, 60369	1.5139
Cisd1, CDGSH iron sulfur domain 1, 110576	1.513
Hsd3b7, hydroxy-delta-5-steroid dehydrogenase, 3 beta- and steroid delta-isomerase 7, 5890494	1.5122
Slc2a2, solute carrier family 2 (facilitated glucose transporter), member 2, 630487	1.5117
Ddc, dopa decarboxylase, 610601	1.5084
Slc2a2, solute carrier family 2 (facilitated glucose transporter), member 2, 2760463	1.5065
Ly6d, lymphocyte antigen 6 complex, locus D, 5560754	1.5031
Aldh1a7, aldehyde dehydrogenase family 1, subfamily A7, 3130288	1.5019
Sc4mol, sterol-C4-methyl oxidase-like, 6420253	1.5002
Hist1h2ak, histone cluster 1, H2ak, 3130609	1.4995
Cyp51, cytochrome P450, family 51, 540020	1.4962
Sc5d, sterol-C5-desaturase (fungal ERG3, delta-5-desaturase) homolog (S. cerevisae), 4150747	1.4952
Pecr, peroxisomal trans-2-enoyl-CoA reductase, 2260561	1.4937
Rarres2, retinoic acid receptor responder (tazarotene induced) 2, 6060367	1.4926
Cyp2e1, cytochrome P450, family 2, subfamily e, polypeptide 1, 6280133	1.4923
Rgn, regucalcin, 3440437	1.4866
LOC100047937, PREDICTED: Mus musculus similar to Aldehyde dehydrogenase 1 family, member L1 (LOC100047937), mRNA., 4890608	1.4863
Rnf125, ring finger protein 125, 4050541	1.4862
Ces1d, carboxylesterase 1D, 670603	1.481

Gamt, guanidinoacetate methyltransferase, 290044	1.4792
Mthfd1, methylenetetrahydrofolate dehydrogenase (NADP+ dependent), methenyltetrahydrofolate cyclohydrolase, formyltetrahydrofolate synthase, 7150484	1.4787
Afm, afamin, 5890706	1.4784
Ttc39c, tetratricopeptide repeat domain 39C, 4670022	1.4757
Rfc4, replication factor C (activator 1) 4, 3940458	1.4749
Tlcd2, TLC domain containing 2, 5900592	1.4718
Apoa5, apolipoprotein A-V, 7380762	1.4717
Cdt1, chromatin licensing and DNA replication factor 1, 1050706	1.4713
Insig1, insulin induced gene 1, 5310343	1.4662
Dcxr, dicarbonyl L-xylulose reductase, 3140274	1.4643
1100001G20Rik, RIKEN cDNA 1100001G20 gene, 1300707	1.4623
Cyp2c55, cytochrome P450, family 2, subfamily c, polypeptide 55, 2360427	1.4618
Fen1, flap structure specific endonuclease 1, 3460037	1.4616
Ephx2, epoxide hydrolase 2, cytoplasmic, 3520491	1.4603
Cyp2f2, cytochrome P450, family 2, subfamily f, polypeptide 2, 6040689	1.4596
Ebpl, emopamil binding protein-like, 110630	1.4572
Sult1a1, sulfotransferase family 1A, phenol-preferring, member 1, 4070215	1.4568
Tecr, trans-2,3-enoyl-CoA reductase, 60220	1.4561
Tecr, trans-2,3-enoyl-CoA reductase, 6620603	1.4551
D0H4S114, DNA segment, human D4S114, 7040243	1.4548
Cdc6, cell division cycle 6 homolog (S. cerevisiae), 2030026	1.4542
Tmie, transmembrane inner ear, 5390632	1.4522
4931406C07Rik, RIKEN cDNA 4931406C07 gene, 630576	1.4501
Lig1, ligase I, DNA, ATP-dependent, 3060767	1.4481
Abat, 4-aminobutyrate aminotransferase, 6020181	1.4472
Haao, 3-hydroxyanthranilate 3,4-dioxygenase, 1740164	1.4452
LOC668837, PREDICTED: Mus musculus similar to ATP synthase, H+ transporting, mitochondrial F0 complex, subunit G (LOC668837), misc RNA., 2690097	1.4445
Paox, polyamine oxidase (exo-N4-amino), 4120470	1.4445
Sec14l2, SEC14-like 2 (S. cerevisiae), 1260075	1.4416
Spp2, secreted phosphoprotein 2, 4860068	1.438
Mrap, melanocortin 2 receptor accessory protein, 5870487	1.4328
Olfml1, olfactomedin-like 1, 4780020	1.4327
Nrn1, neuritin 1, 5050471	1.4321
Gstm4, glutathione S-transferase, mu 4, 2320228	1.4312
Pank1, pantothenate kinase 1, 6290411	1.4311
Spc24, SPC24, NDC80 kinetochore complex component, homolog (S. cerevisiae), 5340398	1.4245
Rrm2, ribonucleotide reductase M2, 5560646	1.4228

Apon, apolipoprotein N, 7400376	1.421
Tmem86b, transmembrane protein 86B, 6250184	1.4204
Hist1h2ah, histone cluster 1, H2ah, 1470341	1.4176
Tdo2, tryptophan 2,3-dioxygenase, 4180187	1.4163
Tecr, trans-2,3-enoyl-CoA reductase, 5900333	1.4162
Pon1, paraoxonase 1, 4390398	1.4152
Baat, bile acid-Coenzyme A: amino acid N-acyltransferase, 2510674	1.4148
Nsdhl, NAD(P) dependent steroid dehydrogenase-like, 2650653	1.4139
Vkorc1, vitamin K epoxide reductase complex, subunit 1, 7650435	1.4119
Selenbp2, selenium binding protein 2, 4920725	1.4108
Slco1b2, solute carrier organic anion transporter family, member 1b2, 1410035	1.4094
Rpa1, replication protein A1, 1340671	1.4085
Atp5a1, ATP synthase, H+ transporting, mitochondrial F1 complex, alpha subunit 1, 6020746	1.4073
Dhcr24, 24-dehydrocholesterol reductase, 6370681	1.4071
Ebpl, emopamil binding protein-like, 5810722	1.4071
Ccl9, chemokine (C-C motif) ligand 9, 7050538	1.4043
Sord, sorbitol dehydrogenase, 5720014	1.4043
Fam73b, family with sequence similarity 73, member B, 1170537	1.4037
Aldh1b1, aldehyde dehydrogenase 1 family, member B1, 2650154	1.4029
Bhmt2, betaine-homocysteine methyltransferase 2, 610576	1.401
Dhdh, dihydrodiol dehydrogenase (dimeric), 830195	1.4003
Hsd17b7, hydroxysteroid (17-beta) dehydrogenase 7, 1070097	1.4001
Fahd1, fumarylacetoacetate hydrolase domain containing 1, 4120692	1.3999
Sgk2, serum/glucocorticoid regulated kinase 2, 3520519	1.3999
Gchfr, GTP cyclohydrolase I feedback regulator, 1340711	1.3989
Slc38a3, solute carrier family 38, member 3, 6100154	1.3958
Reln, reelin, 7610484	1.3955
Tcea3, transcription elongation factor A (SII), 3, 2650372	1.395
Ebp, phenylalkylamine Ca2+ antagonist (emopamil) binding protein, 1990112	1.3942
Cyp27a1, cytochrome P450, family 27, subfamily a, polypeptide 1, 2940170	1.393
Sephs2, selenophosphate synthetase 2, 1770707	1.3923
Rbp4, retinol binding protein 4, plasma, 5420240	1.3914
5730469M10Rik, RIKEN cDNA 5730469M10 gene, 3180386	1.3905
0610007P14Rik, RIKEN cDNA 0610007P14 gene, 1230730	1.3881
Klk1b4, kallikrein 1-related pepidase b4, 1090333	1.3867
Slc25a44, solute carrier family 25, member 44, 5550243	1.3866
Hpgd, hydroxyprostaglandin dehydrogenase 15 (NAD), 2450343	1.3863
Slc38a4, solute carrier family 38, member 4, 5340386	1.386
Lpcat3, lysophosphatidylcholine acyltransferase 3, 2680703	1.384

Ddah1, dimethylarginine dimethylaminohydrolase 1, 2060592	1.3834
Akr1c14, aldo-keto reductase family 1, member C14, 1690632	1.3833
Nfic, nuclear factor I/C, 3840059	1.3818
Mvd, mevalonate (diphospho) decarboxylase, 2100097	1.3818
Entpd5, ectonucleoside triphosphate diphosphohydrolase 5, 2320292	1.3803
Cyp3a11, cytochrome P450, family 3, subfamily a, polypeptide 11, 7320431	1.3801
Cyb5r3, cytochrome b5 reductase 3, 840309	1.3786
Amy1, amylase 1, salivary, 4850164	1.3775
Cyp17a1, cytochrome P450, family 17, subfamily a, polypeptide 1, 670653	1.3773
Hacl1, 2-hydroxyacyl-CoA lyase 1, 7210615	1.3765
Acot3, acyl-CoA thioesterase 3, 2230600	1.3761
Nit2, nitrilase family, member 2, 1090136	1.3759
Ugt2a3, UDP glucuronosyltransferase 2 family, polypeptide A3, 3710040	1.3757
Gstk1, glutathione S-transferase kappa 1, 5890487	1.3709
Mgst3, microsomal glutathione S-transferase 3, 4210619	1.3701
Ces1e, carboxylesterase 1E, 4040259	1.3686
Ttpa, tocopherol (alpha) transfer protein, 7000431	1.3652
Hyi, hydroxypyruvate isomerase homolog (E. coli), 4830576	1.3651
Nsdhl, NAD(P) dependent steroid dehydrogenase-like, 4830717	1.3642
Nsdhl, NAD(P) dependent steroid dehydrogenase-like, 990338	1.3569
Cat, catalase, 4760356	1.3537
Gsta3, glutathione S-transferase, alpha 3, 3780193	1.352
Otc, ornithine transcarbamylase, 4890731	1.3499
Cox7b, cytochrome c oxidase subunit VIIb, 940692	1.3489
Gpld1, glycosylphosphatidylinositol specific phospholipase D1, 6940064	1.3474
Nsdhl, NAD(P) dependent steroid dehydrogenase-like, 5820603	1.3469
Serpinc1, serine (or cysteine) peptidase inhibitor, clade C (antithrombin), member 1, 1690128	1.3458
Pcyt1a, phosphate cytidylyltransferase 1, choline, alpha isoform, 150475	1.3454
Acsm3, acyl-CoA synthetase medium-chain family member 3, 3170270	1.3451
Stard4, StAR-related lipid transfer (START) domain containing 4, 4210288	1.344
Snurf, SNRPN upstream reading frame, 3130246	1.3427
Insig1, insulin induced gene 1, 4920369	1.3405
Pemt, phosphatidylethanolamine N-methyltransferase, 620349	1.3257
Ces2e, carboxylesterase 2E, 780333	1.3239
Pmpcb, peptidase (mitochondrial processing) beta, 450544	1.3196
Hsd11b1, hydroxysteroid 11-beta dehydrogenase 1, 2340301	1.3103

Table 3- Genes downregulated in DZNep treated livers (Sorted via fold change)

Gene name	Fold change
Hbb-b1, hemoglobin, beta adult major chain, 670403	0.4458
Slpi, secretory leukocyte peptidase inhibitor, 2810487	0.4501
Trib3, tribbles homolog 3 (Drosophila), 4280056	0.4549
Hba-a1, hemoglobin alpha, adult chain 1, 2000398	0.4659
Mup21, major urinary protein 21, 3440110	0.4864
Egr1, early growth response 1, 6620079	0.4913
Chac1, ChaC, cation transport regulator-like 1 (E. coli), 540300	0.4955
Ctgf, connective tissue growth factor, 4010082	0.503
Acta2, actin, alpha 2, smooth muscle, aorta, 430068	0.5044
Creld2, cysteine-rich with EGF-like domains 2, 4860079	0.519
Tnfrsf12a, tumor necrosis factor receptor superfamily, member 12a, 1770541	0.5239
Gadd45a, growth arrest and DNA-damage-inducible 45 alpha, 580609	0.536
Gadd45a, growth arrest and DNA-damage-inducible 45 alpha, 3890332	0.5424
Creld2, cysteine-rich with EGF-like domains 2, 290685	0.545
Hspb1, heat shock protein 1, 5670722	0.5471
Emp1, epithelial membrane protein 1, 7160167	0.5534
Hspa8, heat shock protein 8, 2030593	0.571
Ddit3, DNA-damage inducible transcript 3, 1500497	0.573
Syvn1, synovial apoptosis inhibitor 1, synoviolin, 10674	0.5743
Xlr4a, X-linked lymphocyte-regulated 4A, 5870021	0.5867
Herpud1, homocysteine-inducible, endoplasmic reticulum stress-inducible, ubiquitin-like domain member 1, 1050619	0.5935
Herpud1, homocysteine-inducible, endoplasmic reticulum stress-inducible, ubiquitin-like domain member 1, 50129	0.5947
Gtpbp2, GTP binding protein 2, 3850142	0.6031
Cxcl9, chemokine (C-X-C motif) ligand 9, 10598	0.6049
Npy, neuropeptide Y, 160494	0.6069
Plin2, perilipin 2, 5670164	0.6082
Dnajc12, DnaJ (Hsp40) homolog, subfamily C, member 12, 1230441	0.6091
Acta2, actin, alpha 2, smooth muscle, aorta, 2140255	0.6118
Pdlim7, PDZ and LIM domain 7, 6550609	0.6165
Acta2, actin, alpha 2, smooth muscle, aorta, 1850022	0.6281
Cyp2a5, cytochrome P450, family 2, subfamily a, polypeptide 5, 840114	0.633
Oat, ornithine aminotransferase, 4560309	0.6417
Osgin1, oxidative stress induced growth inhibitor 1, 430037	0.6426
Acot1, acyl-CoA thioesterase 1, 450356	0.6447

Tes, testis derived transcript, 7550121	0.6448
Saa3, serum amyloid A 3, 6400719	0.6456
Cd14, CD14 antigen, 6020674	0.6486
Cxcl1, chemokine (C-X-C motif) ligand 1, 3610082	0.6494
Sprr1b, small proline-rich protein 1B, 6450228	0.6503
Zfand2a, zinc finger, AN1-type domain 2A, 1400170	0.6525
Tfrc, transferrin receptor, 620110	0.6552
Slc38a2, solute carrier family 38, member 2, 4640008	0.6559
Ch25h, cholesterol 25-hydroxylase, 1510349	0.6583
S100a8, S100 calcium binding protein A8 (calgranulin A), 1190546	0.661
Cnot3, CCR4-NOT transcription complex, subunit 3; exon 12, 130519	0.6617
Cd9, CD9 antigen, 3990674	0.6626
Ms4a6d, membrane-spanning 4-domains, subfamily A, member 6D, 3180025	0.6634
Ctsj, cathepsin J, 1170041	0.6639
Mt1, metallothionein 1, 5220279	0.6644
Nupr1, nuclear protein 1, 1240424	0.6655
Lgmn, legumain, 1690719	0.6699
Cyp2a5, cytochrome P450, family 2, subfamily a, polypeptide 5, 6350333	0.6701
Hsph1, heat shock 105kDa/110kDa protein 1, 3290270	0.6703
Rhbdf1, rhomboid family 1 (Drosophila), 3840598	0.6715
Tfrc, transferrin receptor, 1780524	0.6718
Foxq1, forkhead box Q1, 1850487	0.6731
Tmsb10, thymosin, beta 10, 5130273	0.6738
P2rx4, purinergic receptor P2X, ligand-gated ion channel 4, 2030484	0.6746
Acta2, actin, alpha 2, smooth muscle, aorta, 3130136	0.677
Arpc1b, actin related protein 2/3 complex, subunit 1B, 3120576	0.6783
Cd63, CD63 antigen, 7050487	0.6799
1500012F01Rik, RIKEN cDNA 1500012F01 gene, 540138	0.6808
Cyp2a5, cytochrome P450, family 2, subfamily a, polypeptide 5, 1500180	0.6821
Tbrg1, transforming growth factor beta regulated gene 1, 1450220	0.6831
Eef2, eukaryotic translation elongation factor 2, 1690070	0.685
Sqstm1, sequestosome 1, 4560619	0.6889
Apcs, serum amyloid P-component, 2320170	0.6895
Lgals3, lectin, galactose binding, soluble 3, 510474	0.6919
Nfkbiz, nuclear factor of kappa light polypeptide gene enhancer in B cells inhibitor, zeta, 6650458	0.692
Mtap7d1, microtubule-associated protein 7 domain containing 1, 670543	0.6975
Ddit3, DNA-damage inducible transcript 3, 1230605	0.6993
Chordc1, cysteine and histidine-rich domain (CHORD)-containing, zinc-binding protein 1, 360725	0.7003
Hist1h1c, histone cluster 1, H1c, 50079	0.701
Fgf21, fibroblast growth factor 21, 6290743	0.7011

Uap1l1, UDP-N-acteylglucosamine pyrophosphorylase 1-like 1, 1410113	0.7022
Zyx, zyxin, 4050400	0.7041
S100a9, S100 calcium binding protein A9 (calgranulin B), 1980603	0.705
S100a6, S100 calcium binding protein A6 (calcyclin), 5080326	0.7052
Ddx17, DEAD (Asp-Glu-Ala-Asp) box polypeptide 17, 6180465	0.7066
Wfdc3, WAP four-disulfide core domain 3, 1570370	0.7067
Srxn1, sulfiredoxin 1 homolog (S. cerevisiae), 2970189	0.707
P2rx4, purinergic receptor P2X, ligand-gated ion channel 4, 3120341	0.7101
Lims2, LIM and senescent cell antigen like domains 2, 3180681	0.7107
Ugt1a6a, UDP glucuronosyltransferase 1 family, polypeptide A6A, 3800446	0.7114
Cd63, CD63 antigen, 5090053	0.7119
Col23a1, procollagen, type 23, alpha 1; 3' UTR, 2030358	0.7133
Dnajb1, DnaJ (Hsp40) homolog, subfamily B, member 1, 4540020	0.7161
Foxa3, forkhead box A3, 5290376	0.7162
Dnttip1, deoxynucleotidyltransferase, terminal, interacting protein 1, 4920072	0.7166
Fndc3b, fibronectin type III domain containing 3B, 7160364	0.7176
Vmp1, vacuole membrane protein 1, 5690327	0.7178
Egfr, epidermal growth factor receptor, 1770292	0.719
Egfr, epidermal growth factor receptor, 4540382	0.7194
Tnfrsf22, tumor necrosis factor receptor superfamily, member 22, 430301	0.7195
Gm11428, predicted gene 11428, 5360370	0.7218
Nrbp2, nuclear receptor binding protein 2, 110561	0.7258
Bag3, BCL2-associated athanogene 3, 6660653	0.7262
Psat1, phosphoserine aminotransferase 1, 2260010	0.727
Tmem86a, transmembrane protein 86A, 2750184	0.7273
Hspa5, heat shock protein 5, 150678	0.7277
Atp2a2, ATPase, Ca++ transporting, cardiac muscle, slow twitch 2, 770349	0.731
Rbpms, RNA binding protein gene with multiple splicing, 4050121	0.7318
Rpl23, ribosomal protein L23, 670176	0.7396
Tmc7, transmembrane channel-like gene family 7, 5910408	0.7398
Stbd1, starch binding domain 1, 1430129	0.7403
Timp1, tissue inhibitor of metalloproteinase 1, 4640215	0.7429
Scara5, scavenger receptor class A, member 5 (putative), 160377	0.747
Dbp, D site albumin promoter binding protein, 3180750	0.796
