# Supplementary material for: A Proof-of-Concept for Epigenetic Therapy of Tissue Fibrosis: Inhibition of Liver Fibrosis Progression by 3-Deazaneplanocin A
Source: Mol Ther. 2017 Jan 4;25(1):218–31. doi: 10.1016/j.ymthe.2016.10.004 (PMC5363305; doi:10.1016/j.ymthe.2016.10.004)
Supplement: Document S2. Article plus Supplemental Information [file mmc2.pdf]

Molecular Therapy
Original Article

A Proof-of-Concept for Epigenetic Therapy of Tissue Fibrosis: Inhibition of Liver Fibrosis Progression by 3-Deazaneplanocin A

Müjdat Zeybel,[1,7,8] Saimir Luli,[1,8] Laura Sabater,[1] Timothy Hardy,[1] Fiona Oakley,[1] Jack Leslie,[1] Agata Page,[1] Eva Moran Salvador,[1] Victoria Sharkey,[1] Hidekazu Tsukamoto,[2,3] David C.K. Chu,[4] Uma Sharan Singh,[4] Mirco Ponzoni,[5] Patrizia Perri,[5] Daniela Di Paolo,[5] Edgar J. Mendivil,[6] Jelena Mann,[1] and Derek A. Mann[1]

[1]Institute of Cellular Medicine, Faculty of Medical Sciences, 4th Floor, William Leech Building, Newcastle University, Framlington Place, Newcastle upon Tyne, NE2 4HH, UK; [2]Southern California Research Center for ALPD and Cirrhosis, Keck School of Medicine, University of Southern California, Los Angeles, CA 90033, USA; [3]Department of Veterans Affairs, Greater Los Angeles Healthcare System, Los Angeles, CA 90033, USA; [4]The University of Georgia College of Pharmacy, Athens, GA 30602, USA; [5]Experimental Therapy Unit, Laboratory of Oncology, Istituto Giannina Gaslini, 16148 Genova, Italy; [6]Department of Molecular Biology and Genomics, Institute for Molecular Biology and Gene Therapy, University of Guadalajara, 44100 Guadalajara, Mexico; [7]School of Medicine, Koc University, 34450 Istanbul, Turkey

The progression of fibrosis in chronic liver disease is dependent upon hepatic stellate cells (HSCs) transdifferentiating to a myofibroblast-like phenotype. This pivotal process is controlled by enzymes that regulate histone methylation and chromatin structure, which may be targets for developing anti-fibrotics. There is limited pre-clinical experimental support for the potential to therapeutically manipulate epigenetic regulators in fibrosis. In order to learn if epigenetic treatment can halt the progression of pre-established liver fibrosis, we treated mice with the histone methyltransferase inhibitor 3-deazaneplanocin A (DZNep) in a naked form or by selectively targeting HSC-derived myofibroblasts via an antibody-liposome-DZNep targeting vehicle. We discovered that DZNep treatment inhibited multiple histone methylation modifications, indicative of a broader specificity than previously reported. This broad epigenetic repression was associated with the suppression of fibrosis progression as assessed both histologically and biochemically. The anti-fibrotic effect of DZNep was reproduced when the drug was selectively targeted to HSC-derived myofibroblasts. Therefore, the in vivo modulation of HSC histone methylation is sufficient to halt progression of fibrosis in the context of continuous liver damage. This discovery and our novel HSC-targeting vehicle, which avoids the unwanted effects of epigenetic drugs on parenchymal liver cells, represents an important proof-of-concept for epigenetic treatment of liver fibrosis.

INTRODUCTION

Fibrosis is a pathology associated with aging, chronic disease, and a variety of connective tissue disorders, including arthritis, systemic scleroderma, and athrofibrosis.[1] The development of fibrosis in a tissue arises from remodelling of connective tissue and the net deposition of a collagen-rich fibril-forming extracellular matrix (ECM). Fibrotic remodelling is often a progressive process culminating in architectural and functional disruption of the affected tissue; in the case of vital tissues, such as the liver, lung, heart, or kidney, fibrosis may lead to organ dysfunction and early mortality. Fibrosis also establishes microenvironments in which cancers are more likely to emerge, an example being liver fibrosis and/or cirrhosis, which is a major risk factor for hepatocellular carcinoma.[2] At the present time, there is a lack of clinically proven effective antifibrotic drugs; the exception being Pirfenidone, now approved for treatment of idiopathic pulmonary fibrosis.[3] There is, therefore, an urgent need to develop novel therapeutic strategies that either suppress fibrosis or promote fibrosis regression.

Myofibroblasts are the major cell type responsible for deposition and maintenance of the fibrotic ECM irrespective of the tissue type or the underlying cause of damage.[4,5] The majority of myofibroblasts are generated locally in response to tissue injury, which usually occurs via the transdifferentiation of precursor cells, such as pericytes or resident fibroblasts, or by the process of epithelial-to-mesenchymal transition.[6,7] A normal wound healing response is self-limiting to enable subsequent tissue regeneration, and this response is associated with clearance of myofibroblasts by apoptosis or reversal of transdifferentiation.[8–10] However, in the context of repeated tissue injury or unresolved chronic inflammation, myofibroblasts persist and establish autocrine signaling pathways that stimulate their survival, proliferation, migration, and continued production of fibrotic ECM. The persistence of tissue myofibroblasts is a common feature of progressive fibrosis and a major driver of disease progression.[4] Furthermore, myofibroblasts within the fibrotic matrix can be

Received 23 March 2016; accepted 21 October 2016;
http://dx.doi.org/10.1016/j.ymthe.2016.10.004.

[8]These authors contributed equally to this work.

Correspondence: Derek A. Mann, Institute of Cellular Medicine, Faculty of Medical Sciences, 4th Floor, William Leech Building, Newcastle University, Framlington Place, Newcastle upon Tyne, NE2 4HH, UK.
E-mail: derek.mann@newcastle.ac.uk

This is an open access article under the CC BY license (http://creativecommons.org/licenses/by/4.0/).

"activated" toward a highly proinflammatory state in response to epithelial stress; this indicates that fibrosis-associated myofibroblasts become orchestrators of inflammation within the diseased tissue.[11] Myofibroblasts are therefore key therapeutic targets in fibrosis, but a major challenge is to identify safe and efficacious drug targets that selectively modulate myofibroblast biology.

Transdifferentiation of resident liver sinusoidal hepatic stellate cells (HSCs) into myofibroblasts is tightly regulated by epigenetic modifications, including relandscaping of the DNA methylome and chromatin remodelling at genes regulating the myofibroblast phenotype.[12–14] EZH2 is the catalytic component of the polycomb repressor 2 complex responsible for methylation of histone 3 lysine 27 (H3K27) and is required for stimulating enrichment of the repressive H3K27me3 mark.[14] Enrichment of H3K27me3 at the PPARγ gene is a fundamental epigenetic modification during HSC transdifferentiation that brings about transcriptional repression of PPARγ; this is an essential step for the cell to acquire its myofibroblastic phenotype. Indeed, forced expression of PPARγ in liver myofibroblasts is sufficient to repress collagen expression and reprogram the HSC phenotype to resemble its precursor quiescent state.[15] Small-molecule inhibitors of EZH2, including GSK126, EPZ-6438, and 3-deazaneplanocin A (DZNep), have been proposed for therapeutic development in cancer.[16–18] We have previously reported in vitro experiments that show that DZNep can irreversibly suppress classic morphological and biochemical changes associated with HSC transdifferentiation.[14] Similar studies in lung myofibroblasts have confirmed that inhibition of EZH2 suppresses their fibrogenic phenotype and decreases collagen production.[19] However, the potential for in vivo inhibition of EZH2 as an antifibrotic strategy has not been determined.

In a well-established in vivo model of HSC transdifferentiation and liver fibrosis, we show that therapeutic administration of DZNep in the context of pre-established liver disease is able to effectively suppress progression of fibrosis despite continued liver damage. Moreover, we have developed an antibody-liposome-targeting vehicle that can specifically deliver encapsulated molecules to liver myofibroblasts.[20] Incorporation of targeting antibody into the surface liposome is a novel approach that further develops liposomal technology that was previously used to deliver agents for experimental treatment of liver fibrosis.[21–24] We demonstrate that in vivo application of this novel targeting approach achieves selective inhibition of the H3K27me3 mark in myofibroblasts and halts progression of fibrosis. Our findings provide an exciting proof-of-concept for the use of emerging epigenetic drugs in the treatment of fibrosis in chronic disease and highlight the therapeutic potential of targeting EZH2 and potentially other profibrogenic histone lysine methyltransferases (HKMTs).

RESULTS

DZNep and Related Purine Analogs Suppress Induction of Type I Collagen Expression

DZNep is a purine nucleoside analog (PNA), which is in a family of compounds, many of which are being used clinically and have been proven to be effective in the treatment of hematological malignancies and autoimmune disorders.[33] In a blinded fashion, we began by determining the ability of several chemically-related PNAs (designated compounds 1–9; Figure S1) to inhibit HSC expression of transcripts for the profibrogenic genes collagen 1A1, αSMA, and TIMP-1. This experiment was carried out in vitro using the widely adopted cell-culture model of HSC transdifferentiation in which freshly isolated primary rodent HSCs are cultured for several days in complete serum-containing media. In this model, HSCs undergo a similar process of transdifferentiation to that described in vivo, which serves as a robust tool for pre-clinical drug discovery.[34] The drugs were added to HSCs that had been freshly isolated from normal rat liver and cultured on plastic in complete serum-containing media for just 1 day, at which point the cells had not yet undergone transdifferentiation. Based on results from our previous studies, the compounds were tested at a single concentration of 1 μg/mL in each case. After a further 6 days, at which point HSCs had adopted the myofibroblast phenotype, cultures were harvested and was RNA isolated for qRT-PCR analyses of gene expression. Compounds 3, 4, 5, and 8 were found to repress collagen 1A1 gene expression; compounds 1 and 3 repressed αSMA gene expression, while compound 2 showed the overall best antifibrogenic performance by inhibiting collagen 1A1, αSMA, and TIMP1 gene expression (Figures 1A–1C). Decoding the experiment revealed that compound 2 was DZNep which, in addition to suppressing expression of all three fibrogenic genes, was confirmed to prevent cultured HSCs from adopting the morphology of an activated myofibroblast (Figure 1D). Furthermore, culturing of quiescent HSCs (qHSCs) in the presence of DZNep resulted in increased apoptosis in day 7 cells (Figures 1E and 1F, left panel) while also reducing proliferation (Figure 1F, right panel). These data indicate that PNAs are a class of compounds with strong potential for antifibrotic activities and confirm that DZNep is a molecule worthy of in vivo investigations.

DZNep Prevents the Progression of Carbon Tetrachloride-Induced Liver Fibrosis

Repetitive exposure of the liver to the hepatotoxin carbon tetrachloride (CCl_4) establishes repeated rounds of liver damage and inflammation, which drives a progressive fibrogenic process chiefly mediated by the activities of myofibroblasts generated from an HSC origin.[10] To determine the in vivo antifibrotic properties of DZNep, adult male C57Bl6 mice were injured with CCl_4 for 2 weeks in order to establish mild fibrosis and were subsequently therapeutically administered DZNep (or vehicle control) over a further 6 weeks while being continually injured with CCl_4 (Figure 2A) Sirius Red staining of liver sections (Figure 2B) and morphometric analysis (Figure 2C) showed the expected progressive accumulation of cross-linked fibril-forming collagens between weeks 2 and 8. Remarkably, this disease progression was attenuated in mice treated with DZNep (Figures 2B and 2C). Staining for αSMA again revealed the expected time-dependent increase in the numbers of myofibroblasts when comparing the 2- and 8-week control groups; however, DZNep treatment prevented this accumulation of scar-forming myofibroblasts (Figures 2D and 2E). There were no significant changes in the number

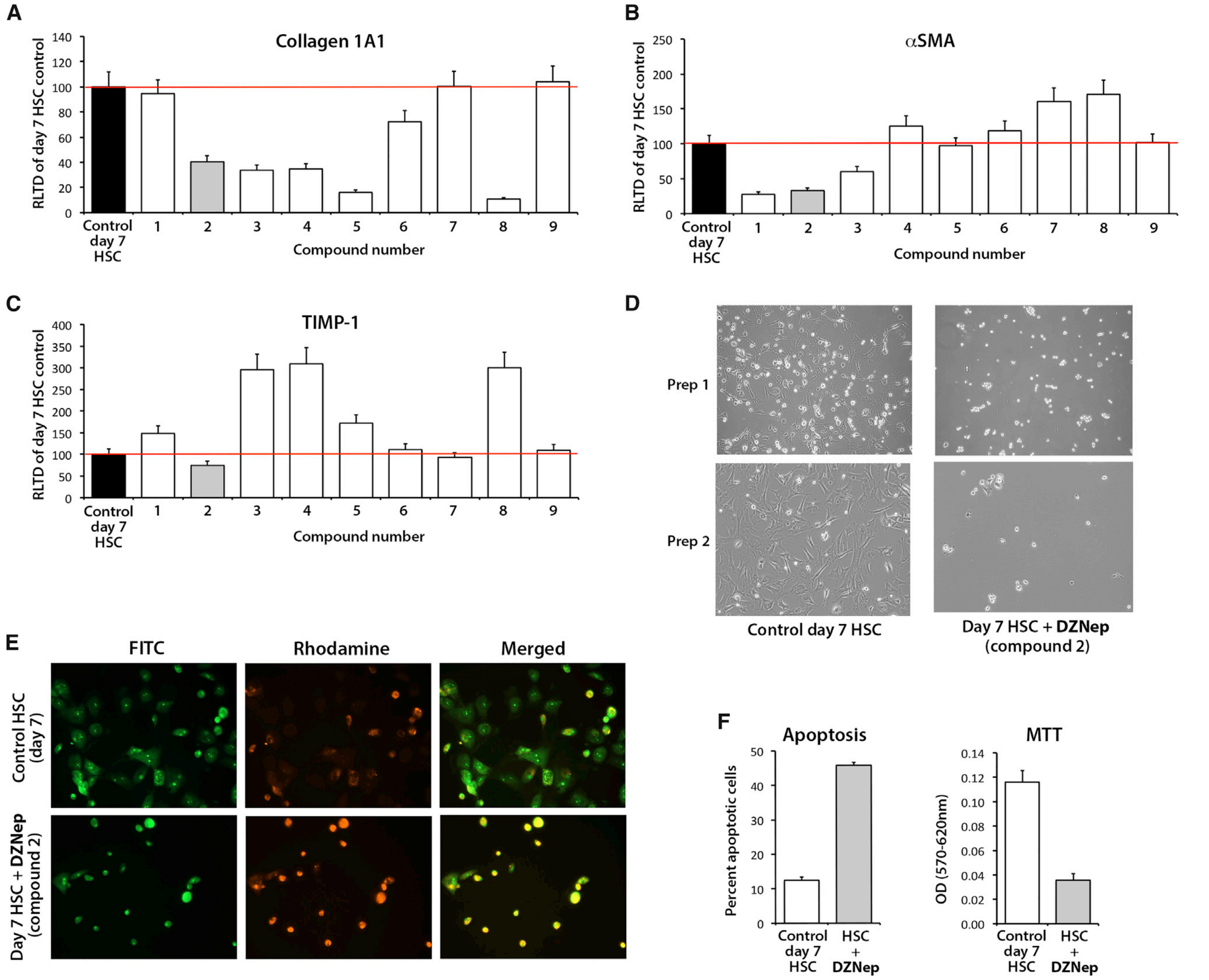

Figure 1. Purine Nucleoside Analogs Demonstrate Varied Ability to Inhibit HSC Transdifferentiation In Vitro

(A–C) Freshly isolated rat HSCs were grown for 7 days in the presence of 1 μg/mL each of a series of chemically related PNAs (designated compounds 1–9). The cells were harvested on day 7, and transcripts for collagen I (A), αSMA (B), and TIMP-1 (C) were quantified by qPCR in at least four separate preparations of HSCs. The best-performing drug across all assays is in gray (compound 2). The line on the bar graphs shows the level of gene expression in control cells. Error bars represent mean ± SEM. RLTD, relative level of transcriptional difference. (D) Representative photomicrographs showing morphological differences between day 7 control-activated HSCs or equivalent culture grown in the presence of 1 μg/mL compound 2 (deazaneplanocin A). (E) Representative FITC (left), rhodamine (middle), and merged (right) fluorescent images of acridine orange-stained day 7 control-activated HSCs or equivalent culture grown in the presence of 1 μg/mL compound 2 (deazaneplanocin A). (F) Graphs showing average percentage of apoptotic cells (left panel) and number of proliferating cells (MTT assay, right panel).

of macrophages observed between the groups (Figure 2F). Quantification of hepatic transcripts at 8 weeks confirmed the anticipated time-dependent increases in expression of fibrogenic collagen 1A1 (Figure 2C), αSMA (Figure 2E), CTGF, TIMP-1 (Figure 2G), IL-6, and transforming growth factor β1 (TGF-β1) (Figure 2H) in control mice. By contrast, in DZNep-treated mice, levels of these transcripts were similar to those measured at 2 weeks, thus reflecting the repressive effect of the drug on accumulation of αSMA+ cells. There were no significant changes in expression of vascular endothelial growth factor (VEGF) or angiopoetin 1 in any of the groups (Figure S2). To ascertain broader effects of DZNep on gene expression, we carried out an unbiased microarray analysis of the hepatic transcriptome comparing the 8-week vehicle control group to the DZNep-treated group (Figure 3). DZNep increased the expression of 248 genes and decreased expression of 108 genes (Tables S1 and S3). The heatmap in Figure 3A shows replicates for the top 15 most upregulated and downregulated genes, while Figures 3B and 3C provide validatory qRT-PCRs for a subset of the downregulated and upregulated genes,

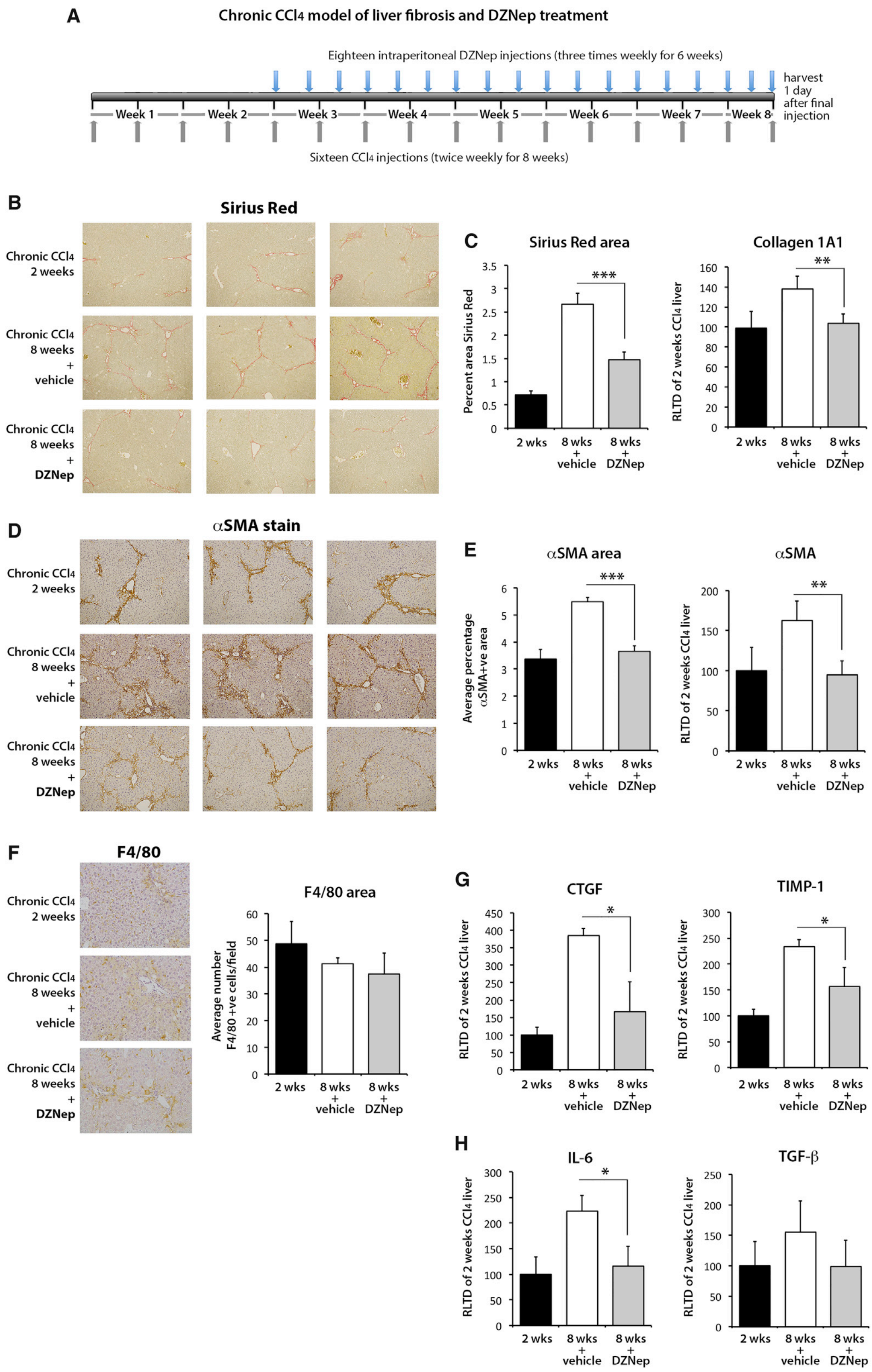

(legend on next page)

respectively. Of particular note, among the downregulated genes were Acta2 (αSMA) and CTGF, confirming the qRT-PCR data; the transcription factor EGR1 was also downregulated, which plays a core role in fibrogenesis by positively regulating the expression of multiple fibrogenic growth factors, including TGF-β1, platelet-derived growth factor (PDGF), and fibroblast growth factor (FGF).[35] Strongly upregulated genes were enriched for those encoding enzymes involved in the metabolism of xenobiotics or bile acids (Cyp2c37, Cyp2c50, Cyp7a1, Cyp8b1, and Inmt), lipids (Acss2 and Thrsp1), iron (Hamp2), and glucose (G6Pc). However, alanine transaminase (ALT) values were not significantly different between the control of DZNep-treated groups, indicating that the drug does not display any obvious hepatotoxicity over and above that caused by CCl_4 and, further, that the observed changes in expression of metabolic genes did not cause any interference with CCl_4-induced liver damage (Figures S3A and S3B). Notably, expression of Cyp2E1, which metabolises CCl_4 in the liver, remained unchanged in this model (Figure S4).

DZNep Acts as a Broad Specificity Inhibitor of Hepatic Histone Methyltransferases

To confirm that the antifibrogenic activity of DZNep was associated with the expected in vivo repression of the H3K27me3 epigenetic mark controlled by EZH2, we carried out western blotting using protein extracts from 8-week vehicle control and DZNep-treated livers. Relative to vehicle controls, a loss of hepatic H3K27me3 was associated with DZNep treatment in the CCl_4 models (Figure 4A). However, we also observed a loss of other histone modifications, including epigenetic marks associated with transcriptional activation (H3K36me3 and H3K4me3) and repression (H3K9me3) (Figure 4A). These data support previous results, indicating a broader effect of DZNep on histone lysine methyltransferase activities than suggested in earlier reports, which claimed specificity of the drug for EZH2.[18] Furthermore, free DZNep was repressing H3K27 trimethyation in multiple hepatic cell types in addition to HSCs, including hepatocytes and cholangiocytes (Figure 4B).

Targeting of DZNep to HSC-Derived Myofibroblasts Inhibits Fibrosis

Given the broad inhibitory effects of DZNep on histone lysine methyltransferases (HKMTs) and the suggestion from hepatic mRNA expression data of potential metabolic effects on hepatocytes, it was plausible that the observed anti-fibrotic activities of the drug might not reflect a direct activity in HSCs. To address this important caveat, we exploited recent advances in liposome-mediated drug delivery, ligand-mediated cell targeting of liposomes, and the specificity of the single chain antibody (ScAb) C1-3 for HSC-derived myofibroblasts.[36,37] C1-3 specifically recognizes synaptophysin, a transmembrane protein that is selectively expressed on HSC-derived myofibroblasts in the diseased liver.[38] We therefore asked if targeted delivery of DZNep to HSC-derived myofibroblasts in C1-3-coated liposomes could bring about a similar therapeutic effect as that observed with free DZNep. Prior to answering this question, we first confirmed the specificity of C1-3-liposome conjugates for liver myofibroblasts. To this end, the cytotoxic drug doxyrubicin (Dox) was incorporated into liposomes as detailed in the Materials and Methods (and summarized in Figure S5). Dox-liposomes were subsequently separated from free Dox by purification over a Sephadex G50 column. Critically, the Dox-liposome complexes were constructed from lipid conjugates, which included a DSPE-PEG_{2000}-MAL group in which the maleimide terminus could be used for coupling to targeting proteins.[26] We exploited this chemistry to couple Dox-liposomes to C1-3 or a control (CSBD9) ScAb, the latter lacking specificity for myofibroblasts. ScAb-Dox-liposomes were then administered to mice undergoing acute injury with a high dose of CCl_4 in which HSCs were stimulated to undergo myofibroblast transdifferentiation (Figure 5A). Relative to control liposomes, C1-3-Dox-liposomes had no effect on CCl_4-induced serum ALT, AST, and ALP values, indicating no obvious impact on hepatocyte death (Figure S6). C1-3-Dox-liposomes had no effect on the number of hepatic macrophages, neutrophils, or proliferating hepatocytes (Figures 5B–5D). In contrast, livers of C1-3-Dox-liposomes had roughly half the number of αSMA+ myofibroblasts compared with controls (Figure 5E), and this was associated with reduced hepatic expression of TGF-β1 (Figure 5F). These data provided us with confidence that C1-3-liposomes provide an effective vehicle for in vivo delivery of encapsulated drugs selectively to HSC-derived myofibroblasts. We next generated C1-3-DZNep-liposomes together with a control vehicle CSBD9-DZNep-liposomes. To determine the in vivo therapeutic potential of DZNep-liposome-C1-3 conjugates, they were administered to mice under a similar experimental CCl_4 therapeutic model as previously described for free DZNep. Mice were initially injured for 2 weeks to establish liver disease, prior to a further 6 weeks of injury, during which time the animals were administered either C1-3-DZNep-liposomes or control CSBD9-DZNep-liposomes (Figure 6A). After 8 weeks, mice were culled, and all liver sections were stained with Sirius red for collagen and by immunohistochemistry for αSMA (Figures 6B and 6C). As shown in Figures 6B and 6C, significantly less

Figure 2. DZNep Prevents Fibrosis Progression in a Chronic Model of CCl_4-Induced Liver Fibrosis

(A) Schematic representation of chronic CCl_4 model of liver fibrosis combined with progressive therapeutic treatment with DZNep. Grey arrows show frequency of CCl_4 injections, whereas blue arrows show DZNep injections. Briefly, liver fibrosis was established for 2 weeks followed by administration of DZNep treatment alongside CCl_4 for a further 6 weeks. (B) Histological sections showing collagen staining (Sirius Red). (C) Graphs showing average percentage area for Sirius Red (left) and mRNA levels of Collagen 1A1 as quantified by qPCR in livers from all animals in the study (right). (D) αSMA staining in three representative control or DZNep-treated animals as well as the animals at the starting point of treatment (2 weeks CCl_4). (E) Graphs showing average percentage area αSMA in all groups (left) and mRNA levels of αSMA as quantified by qPCR in livers (right). (F) Histological sections showing macrophage staining (F4/80, left panels) and graphs showing average number of F4/80 positive cells per field (right panel). (G) mRNA levels for XTΓΦ, TIMΠ−1, (H) IΛ−6, and TΓΦβ1 as quantified by qPCR in livers of all animals. Error bars in relevant panels represent mean ± SEM. *p < 0.05; **p < 0.01; ***p < 0.001.

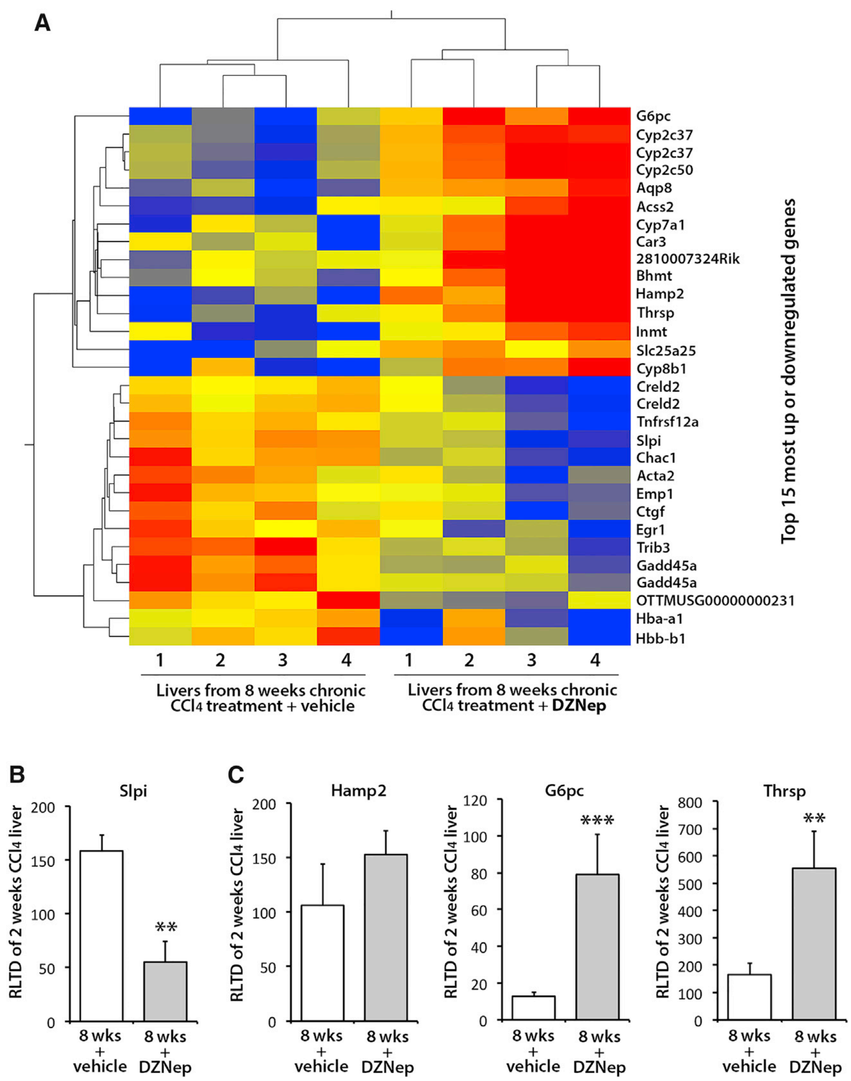

Figure 3. DZNep Alters Expression of Numerous Genes in a Chronic Model of CCl_4-Induced Liver Fibrosis

(A) A heatmap displaying results of microarray carried out using four control and four DZNep-treated livers from a chronic model of CCl_4-induced liver fibrosis. The top 15 most upregulated and downregulated genes are shown. Blue, negative values (i.e., downregulated); red, positive (upregulated); yellow, unchanged. (B) mRNA level of Slpi and (C) Hamp2, G6pc, and Thrsp genes was quantified by qPCR in order to validate the results of microarray. Error bars in relevant panels represent mean ± SEM. $^{**}p < 0.01$; $^{***}p < 0.001$.

fibrotic collagen accumulated in the livers of mice receiving C1-3-DZNep-liposomes compared with those treated with control CSBD9-DZNep-liposomes. This difference in fibrosis was reflected in associated levels of hepatic αSMA, which were lower in livers of C1-3-DZNep-liposome recipients (Figure 6C, right panel). Targetted DZNep also resulted in a significant reduction of collagen 1A1, CTGF, and angiopoetin 1 expression (Figure 6D), while no change was detected in the expression of TIMP1 (Figure S7). We conclude that in vivo targeting of DZNep to HSCs using a C1-3-liposome vehicle leads to a reduction in the number of hepatic myofibroblasts in diseased livers, suppression of collagen deposition, and reduced levels of hepatic fibrosis.

To show specificity of C1-3-DZNep-liposome treatment for hepatic myofibrobalsts, we stained the livers of C1-3-DZNep-liposomes and control CSBD9-DZNep-liposomes for the presence of an H3K27me3 epigenetic mark (Figure 7A). Data showed the absence of H3K27me3 staining only in myofibroblasts in the livers of mice receiving C1-3-DZNep-liposomes, while mice treated with control CSBD9-DZNep-liposomes showed the presence of H3K27me3 in all cell types, including myofibroblasts (Figure 7A). Dual immunofluorescence staining of livers for αSMA and H3K27me3 further confirmed that treatment with C1-3-DZNep-liposomes is associated with selective loss of the epigenetic mark in myofibroblasts (Figure 7B).

DISCUSSION

The concept of epigenetic therapy is now well-established in the field of oncology, with the successful clinical application of inhibitors of DNA methylation (e.g., decitabine) and histone deacetylases (e.g., SAHA) described for many types of cancers.[39] Akin to cancer, fibrosis is a pathology that is associated with dramatic changes in tissue architecture underpinned by alterations in cell differentiation, fate, and function. In particular, the generation, proliferation, and lifespan of myofibroblasts, the major cellular drivers of extracellular matrix deposition, are important determinants of fibrosis progression.[40] Chronic disease is often characterized by an unresolved wound-healing process in which tissue myofibroblasts are continually produced and become highly proliferative, motile, and resistant to apoptosis.[8,40] The behavioral parallels of myofibroblasts to those of neoplastic cells, in particular, their proliferative nature and resistance to apoptosis, have led our group and other investigators to explore the possibility that epigenetic alterations may regulate their phenotype and behavior and, in turn, the course of the fibrogenic process.[41] Transdifferentiation of HSCs represents the major cellular source of myofibroblasts in chronic liver disease and is associated with global changes in gene expression underpinned by re-landscaping of the HSC epigenome, including genome-wide changes in DNA methylation and histone modifications.[12,13,42] Previous in vitro studies and a small number of in vivo studies have demonstrated the ability of pharmacological inhibitors of DNA methylation, histone deacetylation, and histone

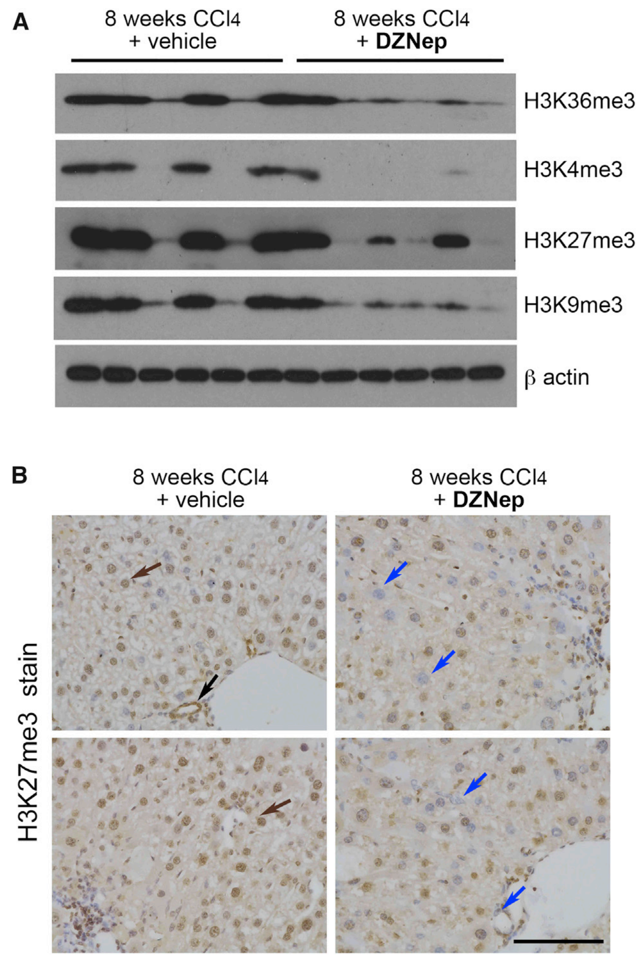

Figure 4. DZNep Inhibition of Histone Methylation Is Not Specific to H3K27me3

(A) 30 μg whole-cell protein from six livers of control animals or six livers from DZNep-treated livers within the chronic model of CCl_4-induced liver fibrosis were immunoblotted for H3K36me3, H3K4me3, H3K27me3, H3K9me3, and β-actin. (B) Histological sections showing H3K27me3 staining in a representative set of vehicle or DZNep-treated chronic CCl_4 livers. Brown arrows show the presence of H3K27me3 staining in hepatocytes and biliary epithelial cells of vehicle-treated fibrotic livers, with H3K27me3 markedly absent in both cell types in DZNep-treated livers.

methylation to suppress in vivo and culture-induced HSC transdifferentiation as well as development of fibrosis.[12,14,43–47] The potential for epigenetic approaches to be exploited for suppressing fibrosis is also supported by in vivo investigations with mice lacking the master epigenetic regulator MeCP2, which is attenuated for fibrosis across multiple tissues, including liver, lung, heart, and retina.[14,48–52] The work we have described in this present study significantly advances these previous investigations by showing that in vivo administration of the epigenetic drug DZNep halts the progression of pre-established experimental liver fibrosis despite sustained liver damage. Remarkably, we were able to demonstrate that this anti-fibrotic activity of DZNep is retained when the drug is selectively targeted to HSC-derived myofibroblasts, thus providing the first proof-of-concept that progressive tissue fibrosis can be therapeutically attenuated via the direct epigenetic manipulation of the myofibroblast.

A major target of DZNep is EZH2, the only HKMT in nature that catalyzes trimethylation at H3K27.[53] EZH2 is aberrantly expressed in numerous cancers, including leukemia, pancreatic ductal adenocarcinoma, and hepatocellular carcinoma, and there are now many pre-clinical studies reporting the inhibitory effects of DZNep on tumor growth.[54,55] Treatment of cells with DZNep results in depletion of EZH2 and, as such, this effect and the associated loss of the H3K27me3 mark is considered to be its major mechanism of anti-tumor activity. However, we report that in vivo administration of DZNep has broader inhibitory effects on histone 3 methylation in the liver, with global diminution of H3K4me3, H3K9me3, and H3K36me3 as well as the anticipated loss of H3K27me3. This non-selective effect of DZNep on histone methylation has previously been described using in vitro cancer cell models where the drug suppressed both repressive and active histone methylation marks.[18] On the one hand, a drug such as DZNep, which has a global impact on histone methylation, may be clinically adventitious since HSC transdifferentiation requires the de novo annotation of multiple repressive and activatory histone methylation marks, thus reflecting the need to repress the expression of genes that promote the adipogenic phenotype of quiescent HSCs while simultaneously programming the transcription of genes that are characteristic of the myofibroblast phenotype.[12–14] As an example, de novo expression of MeCP2 is induced shortly after HSCs are plated into culture and leads to the almost simultaneous de novo expression of EZH2 and ASH1 which, combined, stimulate H3K27me3-mediated repression of anti-fibrogenic PPARγ and H3K4me3-regulated transcription of pro-fibrogenic TGF-β1, TIMP-1, and type I collagen I genes.[13,14] On the other hand, a systemic repression of histone methylation in the context of a long-term therapeutic regimen would be likely to result in unwanted side effects, as might the global loss of EZH2 expression. In this regard, DZNep-mediated suppression of EZH2 has been reported to enhance lipid accumulation and inflammation in high-fat diet models of rodent liver disease.[56] One solution to this problem explored here is the use of a myofibroblast-targeting vehicle to achieve in vivo cell-selective delivery of DZNep. This aim was achieved by initially showing that liposomes coated with the HSCs targeting ScAb C1-3 and loaded with the cytotoxic drug doxyrubicin selectively depleted αSMA+ liver myofibroblasts. We then demonstrated that therapeutic administration of C1-3-coated liposomes carrying DZNep halted fibrosis progression in the CCl_4 model with a similar efficacy to that achieved when administering "naked" DZNep. This approach supports our hypothesis that in vivo therapeutic effects of DZNep are a consequence of direct targeting of epigenetic events in liver myofibroblasts rather than being due to effects on other types of liver cells. We propose that this myofibroblast-selective drug delivery technology may be developed for other therapeutic compounds that have the potential to effect a broad number of cell types and, in particular, for epigenetic drugs that modulate chromatin modifications common to more than one type of liver cell. It is important to note that the basic liposome vehicle we have used lends itself to the incorporation of a wide number of therapeutic molecules, including small drug-like

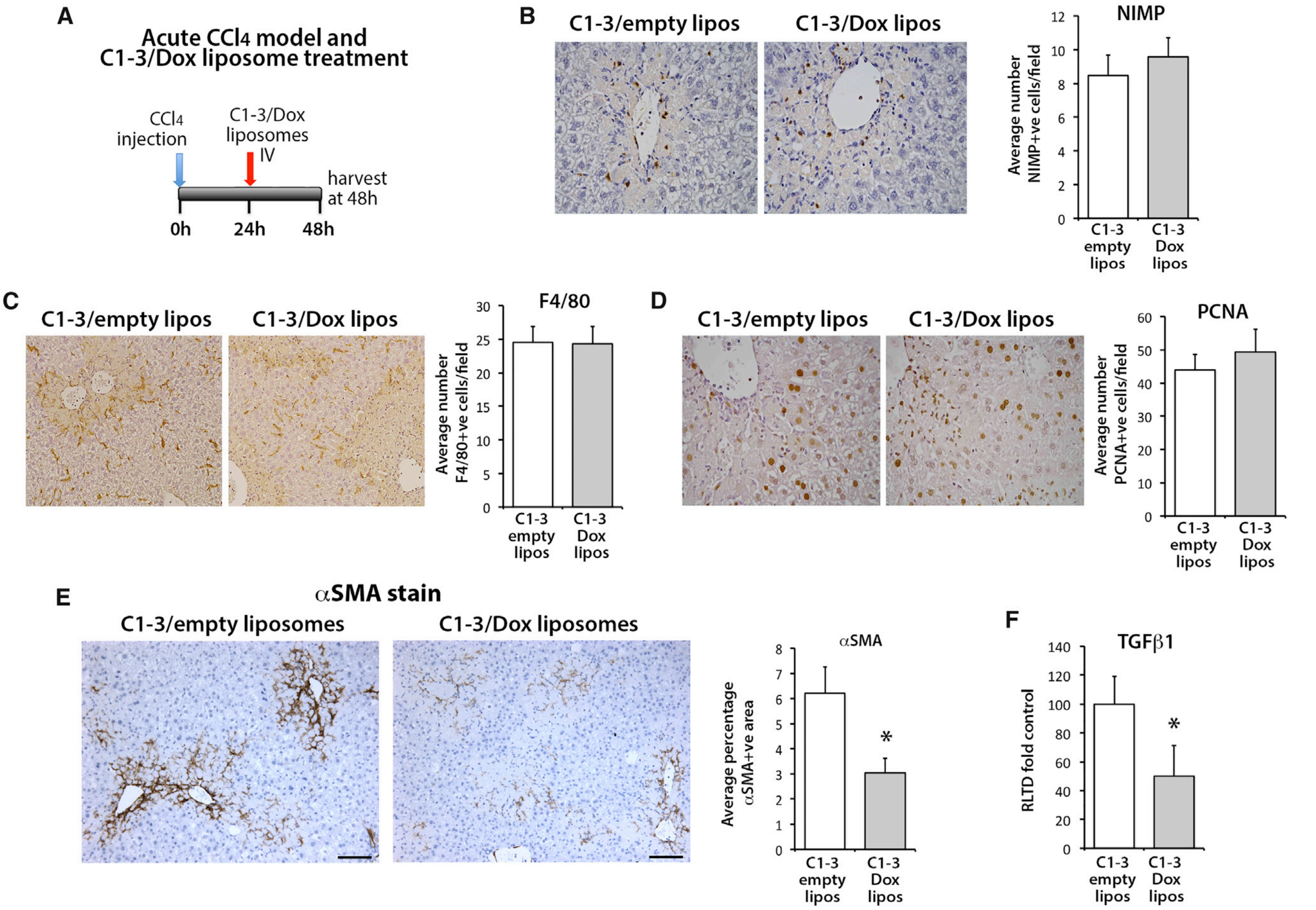

Figure 5. Liposomes Coated with C1-3 ScAb and Loaded with Doxorubicin Significantly Decrease Numbers of Hepatic Myofibroblasts

(A) Schematic representation of acute CCl_4 model of liver fibrosis and therapeutic treatment with C1-3/Dox liposomes. (B) Representative histological sections and graph showing average number of NIMP+ cells (neutrophils), (C) F4/80 (macrophages), and (D) PCNA in control (C1-3/empty liposomes) or C1-3/doxorubicin liposomes treated livers. (E) αSMA staining in representative control (C1-3/empty liposomes) or C1-3/doxorubicin liposome-treated animals and average αSMA positive area in both groups of livers. (F) mRNA levels of TGF-β1 as quantified by qPCR in livers of control (C1-3/empty liposomes) and C1-3/doxorubicin liposome-treated animals. Error bars in relevant panels represent mean ± SEM. *$p < 0.05$.

compounds, modulatory RNAs and DNAs, antibodies, and peptide-based molecules, all of which can theoretically be encapsulated into the vehicle without the need for chemical modifications. A second solution to the problem of specificity of epigenetic therapeutics that is being actively pursued in both academic and industrial groups is the design of highly selective HKMT inhibitors.[57] It is anticipated that we will shortly have available a toolbox of drug-like molecules that have a high degree of specificity for a particular histone-modifying enzyme. As an example, BIX01294 (BIX) is a potent and selective inhibitor of the G9a and GLP members of the SUV39 family of H3K9 methyltransferases that has been shown to attenuate fibrosis in the experimental unilateral ureteral obstruction (UUO) renal disease model.[58]

In summary, we have used the epigenetic inhibitor DZNep in models of pre-established chronic liver disease to establish the concept that progression of liver fibrosis can be manipulated by the pharmacological targeting of epigenetic modifications in myofibroblasts. With this proof-of-concept, there is now a rational basis for the screening of emerging "epi-drugs" as potential anti-fibrotics for either halting or even reversing the fibrotic process in the absence of an effective treatment for the underlying cause of liver damage.

MATERIALS AND METHODS

Ethics

We hold appropriate licenses for animal experiments, which were issued and/or approved by the local ethical committee and UK Home Office.

Cell Culture

HSCs were isolated from normal livers of 350-g adult male Sprague-Dawley rats by sequential perfusion with collagenase and pronase,

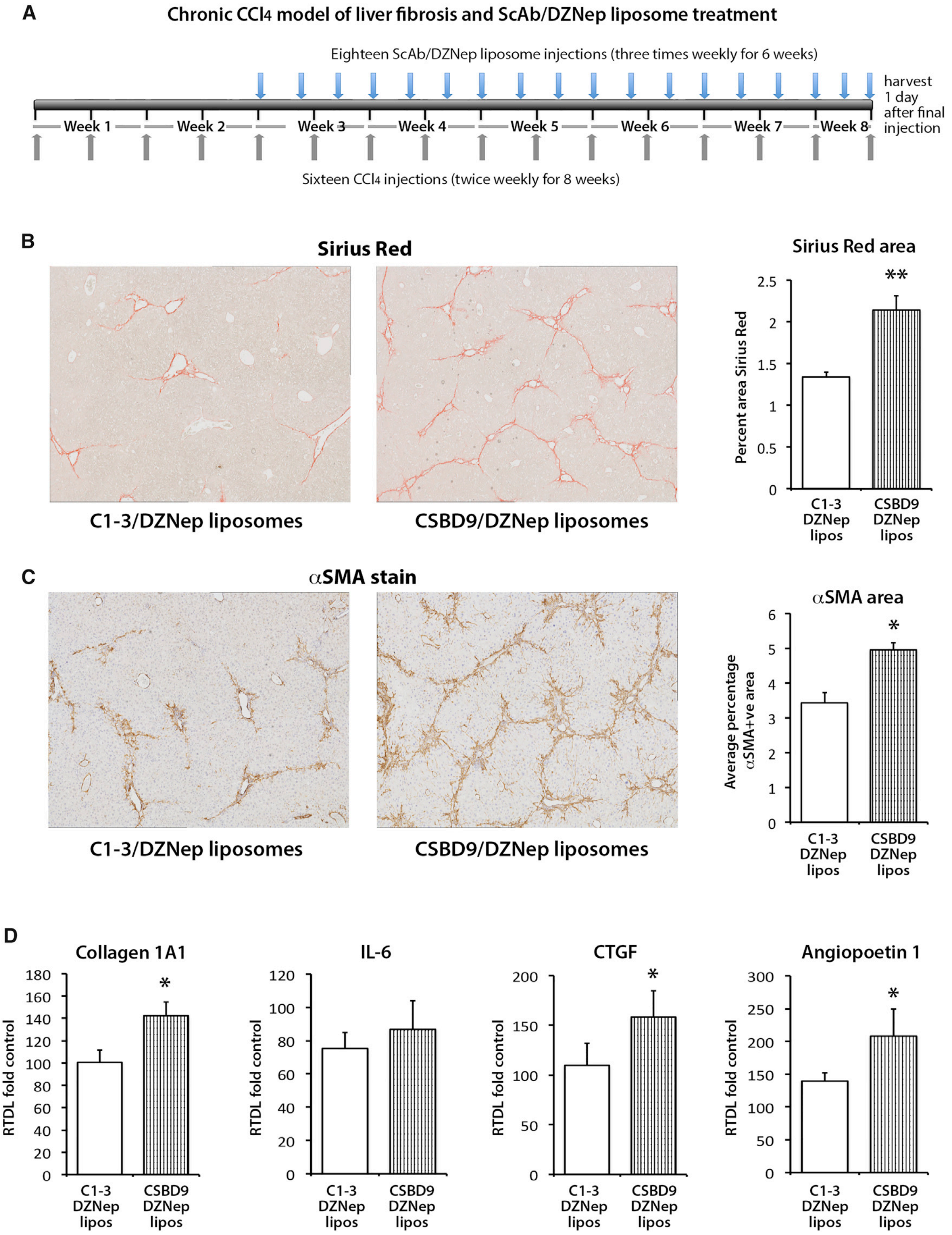

Figure 6. Liposomes Coated with C1-3 ScAb and Loaded with DZNep Significantly Reduce Fibrosis in a Chronic CCl_4 Model of Liver Fibrosis

(A) Schematic representation of the chronic CCl_4 model of liver fibrosis combined with progressive treatment with ScAb/DZNep liposomes. Briefly, liver fibrosis was established for 2 weeks, then the control ScAb CSBD9 or C1-3-coated DZNep-loaded liposomes were administered to animals alongside CCl_4 for a further 6 weeks.

(legend continued on next page)

followed by discontinuous density centrifugation in 11.5% Optiprep (Life Technologies). HSCs were cultured on plastic in DMEM supplemented with penicillin 100 U/mL, streptomycin 100 μg/mL, l-glutamine 2 mmol/L, and 16% fetal calf serum and were maintained at 37°C in an atmosphere of 5% CO_2. Activated HSCs were generated by continuous culture of freshly isolated cells on plastic for 7 days.

Small-Molecule Inhibitors of HSC Activation

Nine proprietary compounds were obtained from D.C.K.C. and tested on day 1 quiescent HSCs in a range of concentrations for their ability to prevent HSC activation in vitro (Figure S1). A concentration of 1 ug/mL was used in the experiments shown. Compounds were applied on quiescent HSCs 12 hr after the isolation, and cells were later harvested at time intervals as indicated.

Histology and/or Immunohistochemistry

Mouse liver tissue was fixed in 10% formalin in PBS, and staining was performed on formalin-fixed paraffin-embedded liver sections. Sirius red staining was performed as previously described.[25] αSMA and H3K27me3 staining was carried out by blocking the endogenous peroxidase activity with 2% hydrogen peroxide in methanol, and then antigen retrieval was achieved using citiric saline antigen unmasking solution (Vector Laboratories). Tissue was blocked using an Avidin/Biotin Blocking Kit (Vector Laboratories) followed by 20% swine serum in PBS and then incubated with primary antibodies; anti-αSMA antibody at 1:1000 (F3777 Sigma) or anti-H3K27 antibody was used at 1:200 (C15410195, diagnode) overnight at 4°C. The next day, slides were PBS washed and then incubated with biotinylated goat anti-fluorescein 1:300 (BA-0601 Vector) or biotinylated swine anti-rabbit 1:200 (eo353 Dako), followed by Vectastain Elite ABC Reagent. Antigens were visualized using a DAB peroxidase substrate kit and counterstained with Mayer's hematoxylin. Slides were imaged using a Nikon ECLIPSE Ni-U (Nikon) microscope, and blinded image analysis of 10 fields at 10× magnification was performed using Nikon Imaging Software Elements Basic Research (NIS-Elements).

For dual αSMA and H3K27me3 staining, slides were treated with citiric saline antigen unmasking solution, then incubated in 0.1% saponin for 10 min. Slides were then PBS washed, blocked with 1× casein and BSA for 60 min, and then incubated with the mouse monoclonal anti-alpha smooth muscle actin FITC conjugated antibodies (Sigma, F3777; dilution factor 1:50) and rabbit anti-H3K27me3 antibodies (dilution factor 1:50) overnight at 4°C. The next morning, slides were PBS washed and incubated with anti-rabbit tetramethylrhodamine (TRITC) secondary Ab (1:100) for 2 hr. Counterstain was performed using 0.3% sudan black in 70% ethanol (EtOH) prior to incubating with DAPI special formulation NucBlue live ready probes reagent (Life Technologies) for 10 min at room temperature. The slides were then mounted with ProLong Gold antifade reagent (Life Technologies). Images were taken using a Leica TCS SP2 UV AOBS MP confocal microscope.

DZNep Liposomal Preparation

Liposomes were synthetized from HSPC:CHE:DSPE-PEG$_{2000}$:DSPE-PEG2$_{000}$-MAL, 2:1:0.06:0.04 molar ratio, respectively. Lipids were dissolved in chloroform at 10 mM and lipids and DZNep were combined at the molar ratio of 11:1. Subsequently, PBS was added, and the mixture was vortexed and then emulsified by sonication for 5 min (200 W) at 4°C using a probe sonicator (Sonicator-ultrasonic liquid processor XL, Misonix). The mixture was then processed by reverse-phase evaporation using a rota-evaporator (Laborota 4000 Heidolph, Asynt) to remove the organic phase by rotary evaporation under a stream of N_2 until the system reverted to the aqueous phase. Following hydration in PBS, liposomes were extruded (LiposoFast-basic extruder, Avestin) through a series of polycarbonate filters of pore size ranging from 400 nm down to 100 nm. Free DZNep was separated from liposomes by passing liposomes over a Sephadex G-50 column pre-equilibrated in PBS. Finally, C1-3 or CSDB9 single-chain variable fragment (ScFvs) are coupled to the maleimide terminus of DSPE-PEG$_{2000}$-MAL using the previously described methods for whole antibodies and for Fab' fragments coupling with slightly modifications.[26] Briefly, to activate the C1-3 and CSBD9 fragments for reactivity toward the maleimide, we utilized 2-iminothiolane (Traut's reagent) to convert exposed amino groups on the antibody into free sulfhydryl groups. A 20:1 mole ratio of 2-iminothiolane to ScFvs and 1 hr of incubation at room temperature with occasional mixing gave optimal ScFv activation. After separation of thiolated ScFvs from iminothiolane with the use of Sephadex G-25 column chromatography, the ScFv was slowly added to the liposomes in the presence of a small magnetic stirring bar. Oxygen was displaced by running a slow stream of nitrogen over the reaction mixture. The tube was capped and sealed with Teflon tape, and the reaction mixture was incubated overnight at room temperature with continuous slow stirring. The resulting immunoliposomes were separated from unreacted ScFvs by chromatography with the use of Sepharose CL-4B, sterilized by filtration through 0.2-μm pore cellulose membranes (Millipore), and stored at 4°C until use. The antibody density was evaluated by BioRad protein assay.

Particle size (in nanometers), polydispersity index (PdI), and zeta potential (Z-potential in megavolts) of liposomal preparations were measured at 25°C using a Malvern Nano ZS90 light scattering apparatus (Malvern Instruments) at a scattering angle of 90°C.[27–31] The physico-chemical features of this novel delivery system are similar to those obtained in our previously published

(B) Histological sections showing collagen staining (Sirius Red) in a representative control or C1-3/DZNep liposome-treated liver. Right panel: graph showing percent positive area stained with Sirius Red. (C) Histological sections showing αSMA staining in a representative control or C1-3/DZNep liposome-treated liver. Right panel: graph showing percent positive area stained with anti αSMA antibody. (D) mRNA levels of Collagen 1A1, IL-6, CTGF, and angiopoetin 1 as quantified by qPCR in livers of control and C1-3/DZNep liposome-treated animals. Error bars in relevant panels represent mean ± SEM. *p < 0.05, **p < 0.01.

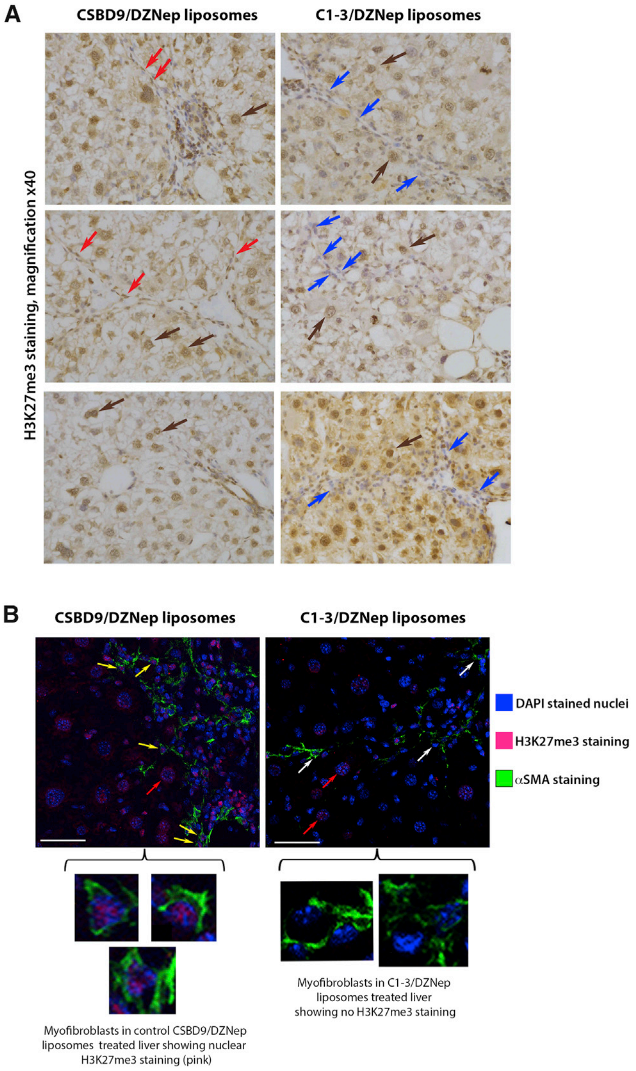

Figure 7. Liposomes Coated with C1-3 ScAb and Loaded with DZNep Reduce the Amount of H3K27me3 Present in Hepatic Myofibroblasts, but Not Hepatocytes

(A) Histological sections showing H3K27me3 staining in a representative set of control CSBD9/DZNep liposomes or C1-3/DZNep liposome-treated chronic CCl_4 livers. Brown arrows, presence of H3K27me3 staining in hepatocytes in both groups; red arrows, presence of H3K27me3 staining in myofibroblasts in CSBD9/DZNep liposome-treated livers; blue arrows, point to myofibroblasts in C1-3/DZNep liposome-treated livers that have lost expression of this hitone mark due to the targeted treatment. (B) Representative images show confocal maximum projections of immunofluorescent-stained liver sections from chronic CCl_4 injured mice treated with CSBD9 or C1-3 coated liposomes, loaded with DZNep at 40× magnification with 1.71 zoom. Sections are stained with DAPI (blue), anti H3K27me3 (red), and anti αSMA (green). Yellow arrows, H3K27me3+ staining (red) in nuclei of αSMA+ cells (green); white arrows, H3K27me3− staining (red) in nuclei of αSMA^+ cells (green); red arrows, H3K27me3+ staining in hepatocytes. Scale bars, 43.63 μM.

studies performed with other encapsulated drugs and nucleic acid liposomal formulations,[27–31] thus indicating a possible clinical translation.

Liver Fibrosis In Vivo Models: CCl_4 and Free Drug DZNep Treatment

Chronic CCl_4 was injected intraperitoneally (i.p.) biweekly at 2 μL (CCl_4/olive oil, 1:3 (v/v))/g/body) for 8 weeks. From 2 weeks onward, in addition to CCl_4, mice received 150 mg/kg DZNep or vehicle triweekly by i.p. injection for a further 6 weeks. Twenty-four hours after the final CCl_4 administration, animals were terminated and liver and serum samples were prepared.

Liver Fibrosis In Vivo Models: CCl_4 and Liposomal DZNep Treatment

Chronic CCl_4 was injected intraperitoneally (i.p.) biweekly at 2 μL (CCl_4/olive oil, 1:3 (v/v))/g/body) for 8 weeks. From 2 weeks onward, in addition to CCl_4, mice received 200 μL of liposomal DZNep preparation coated with C1-3 single chain antibody (ScAb) or CSBD9 control ScAb triweekly by intravenous (i.v.) injection for a further 6 weeks. Twenty-four hours after the final CCl_4 administration, animals were terminated and liver and serum samples were prepared.

RNA Isolation and qRT-PCR

Total RNA was isolated from approximately 200 mg of frozen livers or from $\sim 5 \times 10^6$ cultured cells using the Total RNA Purification Kit (QIAGEN). First-strand complementary DNA was generated by using 1 μg of deoxyribonuclease-treated RNA, 1 μL of random hexamer primer ($p(dN)_6$), and ribonuclease-free water (QIAGEN) heated at 70°C for 5 min and then placed on ice. RNasin (ribonuclease inhibitor), 100 U of Moloney murine leukemia virus reverse transcriptase, 1 × Moloney murine leukemia virus buffer, and 0.4 mmol/L deoxynucleoside triphosphates were added, and the mix was incubated at 42°C for 1 hr. SYBR Green qRT-PCR was performed using the primers listed in Table 1.

qPCR

SYBR Green qRT-PCR reactions were performed in a total volume of 13 μL containing 20 ng of cDNA template, 6.5 μL of SYBR Green JumpStart Taq ReadyMix (Sigma), and 20 pmols of forward and reverse primers (Table 1). The PCR reaction was carried out on a 7500 Fast Real-Time PCR System (Applied Biosystem) with the following parameters: 1 cycle at 95°C for 10 s followed by 40 cycles at 95°C for 10 s, 55°C–60°C (primer pair specific annealing temperature, see Tables 1 and S1) for 30 s, and finally 72°C for 30 s. Melt curve analysis was employed to confirm the presence of a single PCR product. All reactions were normalized to rat β-actin or human GAPDH internal control, and the relative level of transcriptional difference was calculated using the $2^{\Delta\Delta Ct}$ method.

Sodium Dodecyl Sulfate: Polyacrylamide Gel Electrophoresis and Immunoblotting

Whole-cell extracts were prepared, and the protein concentration of samples was determined by using a Bradford DC assay kit (Bio-Rad). Whole-cell extracts from samples of interest were then fractionated by electrophoresis through a 9% sodium dodecyl sulfate-polyacrylamide gel. Gels were run at 100 V for 1.5 hr before

Table 1. qPCR Primers

Gene	Forward and Reverse Primer Pair Sequences	Annealing Temperature (°C)
Mouse Collagen 1A1	TTCACCTACAGCACGCTTGTG	58
	GATGACTGTCTTGCCCCAAGTT	
Mouse CTGF	CAAAGCAGCTGCAAATACCA	58
	GGCCAAATGTGTCTTCCAGT	
Mouse TIMP- 1	GCAACTCGGACCTGGTCATAA	58
	CGGCCCGTGATGAGAAACT	
Mouse IL-6	GAGGATACCACTCCCAACAGACC	58
	AAGTGCATCATCGTTGTTCATACA	
Mouse TGFβ 1	CTCCCGTGGCTTCTAGTGC	58
	GCCTTAGTTTGGACAGGATCTG	
Mouse α SMA	TCAGCGCCTCCAGTTCCT	58
	AAAAAAAACCACGAGTAACAAATCAA	
Mouse Slpi	GTGGAAGGAGGCAAAAATGA	58
	GACATTGGGAGGGTTAAGCA	
Mouse HAMP2	CTGCCTGTCTCCTGCTTCTC	58
	GCAGATGGGGAAGTTGATGT	
Mouse G6pC	TCTGTCCCGGATCTACCTTG	58
	GTAGAATCCAAGCGCGAAAC	
Mouse Thrsp	ACGGAGCCCCTGATCTCTAT	58
	GGCTTCTAGGTCCAGCTCCT	
Mouse GAPDH	GCACAGTCAAGGCCGAGAAT	58
	GCCTTCTCCATGGTGGTGAA	
Mouse angiopoietin 1	AGGCTTGGTTTCTCGTCAGA	56
	TCTGCACAGTCTCGAAATGG	

transfer onto nitrocellulose. After thr blockade of nonspecific protein binding, nitrocellulose blots were incubated for 1 hr with primary antibodies diluted in Tris-buffered saline (TBS)/Tween 20 (0.075%) containing 5% bovine serum albumin. Rabbit polyclonal antibody-recognizing EZH2 was used at 1/500 dilution (Active Motif, catalog no. 39103), H3K27me2 (Abcam ab24684), H3K27me3 (Abcam, ab6002), H3K4me3 (Abcam, ab8580), and β-actin at 1/1000 dilution (Sigma). After incubation with primary antibodies, blots were washed three times in TBS/Tween 20 before incubation for 1 hr in appropriate horseradish peroxidase-conjugated secondary antibody. After extensive washing in TBS/Tween 20, the blots were processed with distilled water for detection of antigen by using the enhanced chemiluminescence system (Amersham Biosciences).

Microarray

Chronic CCl_4 control or DZNep-treated livers (as outlined in the liver fibrosis in vivo models) were used to prepare total RNA, which was utilized for ILMR8 Illumina service MouseRef-8 v2.0 Expression BeadChip. Analysis of microarray data was performed using R from Bioconductor, which has superior normalization specific for illumina arrays (http://www.bioconductor.org/packages/2.10/bioc/html/lumi.html). Following this stage, Rank Prod was used to generate a list of differentially expressed genes. (http://www.bioconductor.org/packages/2.10/bioc/html/RankProd.html).

MTT Assay

Solutions of 3-[4, 5-dimethylthiazol-2-yl]-2, 5-diphenyl tetrazolium bromide (MTT) were dissolved in cell culture medium. HSC treated ± DZNep were seeded at 1×10^5/mL per well in a 12-well tissue culture plate (Greiner) and cultured for 7 days. Cells were PBS washed and then serum starved overnight in 0.1% serum containing media. 100 μL stock of MTT (Sigma) salt (final concentration of 0.5 mg/mL in DPBS) was added to each well for 2 hr at 37°C. Formazan crystals formed were solubilized using 800 μL isopropanol with gentle agitation at room temperature; 200 μL from each well was transferred into a flat bottomed 96 well dish and then quantified using a spectrophotometric plate reader at 570 nm/620 nm and analyzed with SoftMax Pro software.

Quantification of Apoptosis

Apoptotic cells were identified and counted by acridine orange staining and counted as previously described.[32] Acridine orange emits green fluorescence when bound to double-stranded DNA (dsDNA) in the FITC channel and red fluorescence when bound to single-stranded DNA (ssDNA) or RNA in the Rhodamine channel. Images were taken at 10× magnification using a Zeiss axio observer D.1.

Statistical Analysis

Data are expressed as mean ± SEM. All p values were calculated using a two-tailed paired or unpaired Student t test. Statistically significant data are represented in figures where $^*p < 0.05$, $^{**}p < 0.01$, and $^{***}p < 0.001$, respectively.

SUPPLEMENTAL INFORMATION

Supplemental Information includes seven figures and two tables and can be found with this article online at http://dx.doi.org/10.1016/j.ymthe.2016.10.004.

AUTHOR CONTRIBUTIONS

M.Z. and S.L. performed the majority of the laboratory based experiments, data collection, and analyses with technical support from T.H., L.S., F.O., A.P., V.S., and J.L. M.P., P.P., and D.D.P. carried out work generating the ScAb-liposome targeting vehicles. D.C.K.U. and U.S.S. were responsible for the synthesis of DZNep and chemical derivatives. E.J.M. performed western blot investigations on the effects of DZNep on histone methylation modifications. J.M. was responsible for the in vivo therapeutic models. H.T., J.M., and D.A.M. obtained funding support for the study and conceived the work. J.M. and D.A.M. were responsible for experimental design. D.A.M. wrote the manuscript with assistance from J.M. All authors read and contributed to editing of the final submitted manuscript.

CONFLICTS OF INTEREST

The authors who have taken part in this study declare that they do not have anything to disclose regarding funding or conflict of interest with respect to this manuscript.

ACKNOWLEDGMENTS

This work was funded by the UK Medical Research Council (grant MR/K10019494/1 to D.A.M.), the Wellcome Trust (WT086755MA to D.A.M.), the cross-council Lifelong Health and Wellbeing initiative (managed by UK Medical Research Council, award reference L016354), the National Institute on Alcohol Abuse and Alcoholism (grant UA1AA018663 to H.T., D.A.M., and J.M. and R24AA012885 [Non-Parenchymal Liver Cell Core] to H.T.). M.Z. was funded by the EASL Physician Scientist Sheila Sherlock Fellowship, the European Commission, Horizon 2020, and the Marie Sklodowska Curie Individual Fellowship.

REFERENCES

1. Rockey, D.C., Bell, P.D., and Hill, J.A. (2015). Fibrosis–A Common Pathway to Organ Injury and Failure. N. Engl. J. Med. *373*, 96.
2. Llovet, J.M., and Bruix, J. (2008). Novel advancements in the management of hepatocellular carcinoma in 2008. J. Hepatol. *48* (*Suppl 1*), S20–S37.
3. Azuma, A., Nukiwa, T., Tsuboi, E., Suga, M., Abe, S., Nakata, K., Taguchi, Y., Nagai, S., Itoh, H., Ohi, M., et al. (2005). Double-blind, placebo-controlled trial of pirfenidone in patients with idiopathic pulmonary fibrosis. Am. J. Respir. Crit. Care Med. *171*, 1040–1047.
4. Gabbiani, G. (2003). The myofibroblast in wound healing and fibrocontractive diseases. J. Pathol. *200*, 500–503.
5. Hinz, B., Phan, S.H., Thannickal, V.J., Galli, A., Bochaton-Piallat, M.L., and Gabbiani, G. (2007). The myofibroblast: one function, multiple origins. Am. J. Pathol. *170*, 1807–1816.
6. Schrimpf, C., and Duffield, J.S. (2011). Mechanisms of fibrosis: the role of the pericyte. Curr. Opin. Nephrol. Hypertens. *20*, 297–305.
7. Guarino, M., Tosoni, A., and Nebuloni, M. (2009). Direct contribution of epithelium to organ fibrosis: epithelial-mesenchymal transition. Hum. Pathol. *40*, 1365–1376.
8. Elsharkawy, A.M., Oakley, F., and Mann, D.A. (2005). The role and regulation of hepatic stellate cell apoptosis in reversal of liver fibrosis. Apoptosis *10*, 927–939.
9. Kisseleva, T., Cong, M., Paik, Y., Scholten, D., Jiang, C., Benner, C., Iwaisako, K., Moore-Morris, T., Scott, B., Tsukamoto, H., et al. (2012). Myofibroblasts revert to an inactive phenotype during regression of liver fibrosis. Proc. Natl. Acad. Sci. USA *109*, 9448–9453.
10. Mederacke, I., Hsu, C.C., Troeger, J.S., Huebener, P., Mu, X., Dapito, D.H., Pradere, J.P., and Schwabe, R.F. (2013). Fate tracing reveals hepatic stellate cells as dominant contributors to liver fibrosis independent of its aetiology. Nat. Commun. *4*, 2823.
11. Suwara, M.I., Green, N.J., Borthwick, L.A., Mann, J., Mayer-Barber, K.D., Barron, L., Corris, P.A., Farrow, S.N., Wynn, T.A., Fisher, A.J., and Mann, D.A. (2014). IL-1α released from damaged epithelial cells is sufficient and essential to trigger inflammatory responses in human lung fibroblasts. Mucosal Immunol. *7*, 684–693.
12. Page, A., Paoli, P., Moran Salvador, E., White, S., French, J., and Mann, J. (2016). Hepatic stellate cell transdifferentiation involves genome-wide remodeling of the DNA methylation landscape. J. Hepatol. *64*, 661–673.
13. Perugorria, M.J., Wilson, C.L., Zeybel, M., Walsh, M., Amin, S., Robinson, S., White, S.A., Burt, A.D., Oakley, F., Tsukamoto, H., et al. (2012). Histone methyltransferase ASH1 orchestrates fibrogenic gene transcription during myofibroblast transdifferentiation. Hepatology *56*, 1129–1139.
14. Mann, J., Chu, D.C., Maxwell, A., Oakley, F., Zhu, N.L., Tsukamoto, H., and Mann, D.A. (2010). MeCP2 controls an epigenetic pathway that promotes myofibroblast transdifferentiation and fibrosis. Gastroenterology. *138*, 705–714, 714.e1–e4.
15. Hazra, S., Xiong, S., Wang, J., Rippe, R.A., Krishna, V., Chatterjee, K., and Tsukamoto, H. (2004). Peroxisome proliferator-activated receptor gamma induces a phenotypic switch from activated to quiescent hepatic stellate cells. J. Biol. Chem. *279*, 11392–11401.
16. McCabe, M.T., Ott, H.M., Ganji, G., Korenchuk, S., Thompson, C., Van Aller, G.S., Liu, Y., Graves, A.P., Della Pietra, A., 3rd, Diaz, E., et al. (2012). EZH2 inhibition as a therapeutic strategy for lymphoma with EZH2-activating mutations. Nature *492*, 108–112.
17. Knutson, S.K., Kawano, S., Minoshima, Y., Warholic, N.M., Huang, K.C., Xiao, Y., Kadowaki, T., Uesugi, M., Kuznetsov, G., Kumar, N., et al. (2014). Selective inhibition of EZH2 by EPZ-6438 leads to potent antitumor activity in EZH2-mutant non-Hodgkin lymphoma. Mol. Cancer Ther. *13*, 842–854.
18. Miranda, T.B., Cortez, C.C., Yoo, C.B., Liang, G., Abe, M., Kelly, T.K., Marquez, V.E., and Jones, P.A. (2009). DZNep is a global histone methylation inhibitor that reactivates developmental genes not silenced by DNA methylation. Mol. Cancer Ther. *8*, 1579–1588.
19. Coward, W.R., Feghali-Bostwick, C.A., Jenkins, G., Knox, A.J., and Pang, L. (2014). A central role for G9a and EZH2 in the epigenetic silencing of cyclooxygenase-2 in idiopathic pulmonary fibrosis. FASEB J. *28*, 3183–3196.
20. Luli, S., Di Paolo, D., Perri, P., Brignole, C., Hill, S.J., Brown, H., Leslie, J., Marshall, H.L., Wright, M.C., Mann, D.A., et al. (2016). A new fluorescence-based optical imaging method to non-invasively monitor hepatic myofibroblasts in vivo. J. Hepatol. *65*, 75–83.
21. Sato, Y., Murase, K., Kato, J., Kobune, M., Sato, T., Kawano, Y., Takimoto, R., Takada, K., Miyanishi, K., Matsunaga, T., et al. (2008). Resolution of liver cirrhosis using vitamin A-coupled liposomes to deliver siRNA against a collagen-specific chaperone. Nat. Biotechnol. *26*, 431–442.
22. Li, F., Li, Q.H., Wang, J.Y., Zhan, C.Y., Xie, C., and Lu, W.Y. (2012). Effects of interferon-gamma liposomes targeted to platelet-derived growth factor receptor-beta on hepatic fibrosis in rats. J. Control. Release *159*, 261–270.
23. Du, S.L., Pan, H., Lu, W.Y., Wang, J., Wu, J., and Wang, J.Y. (2007). Cyclic Arg-Gly-Asp peptide-labeled liposomes for targeting drug therapy of hepatic fibrosis in rats. J. Pharmacol. Exp. Ther. *322*, 560–568.
24. Chai, N.L., Fu, Q., Shi, H., Cai, C.H., Wan, J., Xu, S.P., and Wu, B.Y. (2012). Oxymatrine liposome attenuates hepatic fibrosis via targeting hepatic stellate cells. World J. Gastroenterol. *18*, 4199–4206.
25. Wright, M.C., Issa, R., Smart, D.E., Trim, N., Murray, G.I., Primrose, J.N., Arthur, M.J., Iredale, J.P., and Mann, D.A. (2001). Gliotoxin stimulates the apoptosis of human and rat hepatic stellate cells and enhances the resolution of liver fibrosis in rats. Gastroenterology *121*, 685–698.
26. Pastorino, F., Stuart, D., Ponzoni, M., and Allen, T.M. (2001). Targeted delivery of antisense oligonucleotides in cancer. J. Control. Release *74*, 69–75.
27. Loi, M., Di Paolo, D., Soster, M., Brignole, C., Bartolini, A., Emionite, L., Sun, J., Becherini, P., Curnis, F., Petretto, A., et al. (2013). Novel phage display-derived neuroblastoma-targeting peptides potentiate the effect of drug nanocarriers in preclinical settings. J. Control. Release *170*, 233–241.
28. Pastorino, F., Brignole, C., Marimpietri, D., Sapra, P., Moase, E.H., Allen, T.M., and Ponzoni, M. (2003). Doxorubicin-loaded Fab' fragments of anti-disialoganglioside immunoliposomes selectively inhibit the growth and dissemination of human neuroblastoma in nude mice. Cancer Res. *63*, 86–92.
29. Di Paolo, D., Pastorino, F., Zuccari, G., Caffa, I., Loi, M., Marimpietri, D., Brignole, C., Perri, P., Cilli, M., Nico, B., et al. (2013). Enhanced anti-tumor and anti-angiogenic efficacy of a novel liposomal fenretinide on human neuroblastoma. J. Control. Release *170*, 445–451.
30. Di Paolo, D., Ambrogio, C., Pastorino, F., Brignole, C., Martinengo, C., Carosio, R., Loi, M., Pagnan, G., Emionite, L., Cilli, M., et al. (2011). Selective therapeutic targeting of the anaplastic lymphoma kinase with liposomal siRNA induces apoptosis and inhibits angiogenesis in neuroblastoma. Mol. Ther. *19*, 2201–2212.
31. Di Paolo, D., Brignole, C., Pastorino, F., Carosio, R., Zorzoli, A., Rossi, M., Loi, M., Pagnan, G., Emionite, L., Cilli, M., et al. (2011). Neuroblastoma-targeted nanoparticles entrapping siRNA specifically knockdown ALK. Mol. Ther. *19*, 1131–1140.
32. Oakley, F., Meso, M., Iredale, J.P., Green, K., Marek, C.J., Zhou, X., May, M.J., Millward-Sadler, H., Wright, M.C., and Mann, D.A. (2005). Inhibition of inhibitor of kappaB kinases stimulates hepatic stellate cell apoptosis and accelerated recovery from rat liver fibrosis. Gastroenterology *128*, 108–120.
33. Tam, E.K., Nguyen, T.M., Lim, C.Z., Lee, P.L., Li, Z., Jiang, X., Santhanakrishnan, S., Tan, T.W., Goh, Y.L., Wong, S.Y., et al. (2015). 3-Deazaneplanocin A and neplanocin A analogues and their effects on apoptotic cell death. ChemMedChem *10*, 173–182.

34. Liedtke, C., Luedde, T., Sauerbruch, T., Scholten, D., Streetz, K., Tacke, F., Tolba, R., Trautwein, C., Trebicka, J., and Weiskirchen, R. (2013). Experimental liver fibrosis research: update on animal models, legal issues and translational aspects. Fibrogenesis Tissue Repair *6*, 19.

35. Pritchard, M.T., and Nagy, L.E. (2005). Ethanol-induced liver injury: potential roles for egr-1. Alcohol. Clin. Exp. Res. *29* (11, Suppl), 146S–150S.

36. Pastorino, F., Marimpietri, D., Brignole, C., Di Paolo, D., Pagnan, G., Daga, A., Piccardi, F., Cilli, M., Allen, T.M., and Ponzoni, M. (2007). Ligand-targeted liposomal therapies of neuroblastoma. Curr. Med. Chem. *14*, 3070–3078.

37. Douglass, A., Wallace, K., Parr, R., Park, J., Durward, E., Broadbent, I., Barelle, C., Porter, A.J., and Wright, M.C. (2008). Antibody-targeted myofibroblast apoptosis reduces fibrosis during sustained liver injury. J. Hepatol. *49*, 88–98.

38. Elrick, L.J., Leel, V., Blaylock, M.G., Duncan, L., Drever, M.R., Strachan, G., Charlton, K.A., Koruth, M., Porter, A.J., and Wright, M.C. (2005). Generation of a monoclonal human single chain antibody fragment to hepatic stellate cells–a potential mechanism for targeting liver anti-fibrotic therapeutics. J. Hepatol. *42*, 888–896.

39. Rius, M., and Lyko, F. (2012). Epigenetic cancer therapy: rationales, targets and drugs. Oncogene *31*, 4257–4265.

40. Hinz, B. (2007). Formation and function of the myofibroblast during tissue repair. J. Invest. Dermatol. *127*, 526–537.

41. Mann, J., and Mann, D.A. (2013). Epigenetic regulation of wound healing and fibrosis. Curr. Opin. Rheumatol. *25*, 101–107.

42. Page, A., Paoli, P.P., Hill, S.J., Howarth, R., Wu, R., Kweon, S.M., French, J., White, S., Tsukamoto, H., Mann, D.A., and Mann, J. (2015). Alcohol directly stimulates epigenetic modifications in hepatic stellate cells. J. Hepatol. *62*, 388–397.

43. Mann, J., Oakley, F., Akiboye, F., Elsharkawy, A., Thorne, A.W., and Mann, D.A. (2007). Regulation of myofibroblast transdifferentiation by DNA methylation and MeCP2: implications for wound healing and fibrogenesis. Cell Death Differ. *14*, 275–285.

44. Niki, T., Rombouts, K., De Bleser, P., De Smet, K., Rogiers, V., Schuppan, D., Yoshida, M., Gabbiani, G., and Geerts, A. (1999). A histone deacetylase inhibitor, trichostatin A, suppresses myofibroblastic differentiation of rat hepatic stellate cells in primary culture. Hepatology *29*, 858–867.

45. Park, K.C., Park, J.H., Jeon, J.Y., Kim, S.Y., Kim, J.M., Lim, C.Y., Lee, T.H., Kim, H.K., Lee, H.G., Kim, S.M., et al. (2014). A new histone deacetylase inhibitor improves liver fibrosis in BDL rats through suppression of hepatic stellate cells. Br. J. Pharmacol. *171*, 4820–4830.

46. Liu, Y., Wang, Z., Wang, J., Lam, W., Kwong, S., Li, F., Friedman, S.L., Zhou, S., Ren, Q., Xu, Z., et al. (2013). A histone deacetylase inhibitor, largazole, decreases liver fibrosis and angiogenesis by inhibiting transforming growth factor-β and vascular endothelial growth factor signalling. Liver Int. *33*, 504–515.

47. Mannaerts, I., Nuytten, N.R., Rogiers, V., Vanderkerken, K., van Grunsven, L.A., and Geerts, A. (2010). Chronic administration of valproic acid inhibits activation of mouse hepatic stellate cells in vitro and in vivo. Hepatology *51*, 603–614.

48. He, S., Barron, E., Ishikawa, K., Nazari Khanamiri, H., Spee, C., Zhou, P., Kase, S., Wang, Z., Dustin, L.D., and Hinton, D.R. (2015). Inhibition of DNA Methylation and Methyl-CpG-Binding Protein 2 Suppresses RPE Transdifferentiation: Relevance to Proliferative Vitreoretinopathy. Invest. Ophthalmol. Vis. Sci. *56*, 5579–5589.

49. Feng, Y., Huang, W., Wani, M., Yu, X., and Ashraf, M. (2014). Ischemic preconditioning potentiates the protective effect of stem cells through secretion of exosomes by targeting Mecp2 via miR-22. PLoS ONE *9*, e88685.

50. Mayer, S.C., Gilsbach, R., Preissl, S., Monroy Ordonez, E.B., Schnick, T., Beetz, N., Lother, A., Rommel, C., Ihle, H., Bugger, H., et al. (2015). Adrenergic Repression of the Epigenetic Reader MeCP2 Facilitates Cardiac Adaptation in Chronic Heart Failure. Circ. Res. *117*, 622–633.

51. Tao, H., Yang, J.J., Hu, W., Shi, K.H., Deng, Z.Y., and Li, J. (2016). MeCP2 regulation of cardiac fibroblast proliferation and fibrosis by down-regulation of DUSP5. Int. J. Biol. Macromol. *82*, 68–75.

52. Hu, B., Gharaee-Kermani, M., Wu, Z., and Phan, S.H. (2011). Essential role of MeCP2 in the regulation of myofibroblast differentiation during pulmonary fibrosis. Am. J. Pathol. *178*, 1500–1508.

53. Tan, J.Z., Yan, Y., Wang, X.X., Jiang, Y., and Xu, H.E. (2014). EZH2: biology, disease, and structure-based drug discovery. Acta Pharmacol. Sin. *35*, 161–174.

54. Völkel, P., Dupret, B., Le Bourhis, X., and Angrand, P.O. (2015). Diverse involvement of EZH2 in cancer epigenetics. Am. J. Transl. Res. *7*, 175–193.

55. Kondo, Y. (2014). Targeting histone methyltransferase EZH2 as cancer treatment. J. Biochem. *156*, 249–257.

56. Vella, S., Gnani, D., Crudele, A., Ceccarelli, S., De Stefanis, C., Gaspari, S., Nobili, V., Locatelli, F., Marquez, V.E., Rota, R., and Alisi, A. (2013). EZH2 down-regulation exacerbates lipid accumulation and inflammation in in vitro and in vivo NAFLD. Int. J. Mol. Sci. *14*, 24154–24168.

57. McGrath, J., and Trojer, P. (2015). Targeting histone lysine methylation in cancer. Pharmacol. Ther. *150*, 1–22.

58. Irifuku, T., Doi, S., Sasaki, K., Doi, T., Nakashima, A., Ueno, T., Yamada, K., Arihiro, K., Kohno, M., and Masaki, T. (2015). Inhibition of H3K9 histone methyltransferase G9a attenuates renal fibrosis and retains klotho expression. Kidney Int. *89*, 147–157.

YMTHE, Volume *25*

Supplemental Information

A Proof-of-Concept for Epigenetic Therapy of Tissue Fibrosis: Inhibition of Liver Fibrosis Progression by 3-Deazaneplanocin A

Müjdat Zeybel, Saimir Luli, Laura Sabater, Timothy Hardy, Fiona Oakley, Jack Leslie, Agata Page, Eva Moran Salvador, Victoria Sharkey, Hidekazu Tsukamoto, David C.K. Chu, Uma Sharan Singh, Mirco Ponzoni, Patrizia Perri, Daniela Di Paolo, Edgar J. Mendivil, Jelena Mann, and Derek A. Mann

Supplementary material for manuscript "**A Proof-of-Concept for Epigenetic Therapy of Chronic Liver Disease: Inhibition of Fibrosis Progression by 3-Deazanoplanocin A (DZNep)**"

Compound. No.	Structure
**1.**	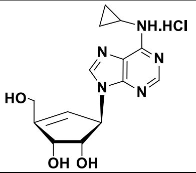
**2.**	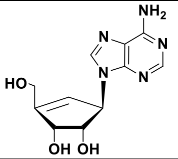
**3.**	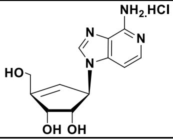
**4.**	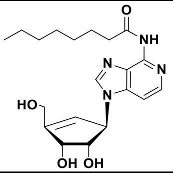
**5.**	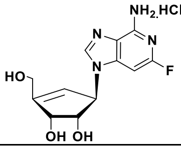
**6.**	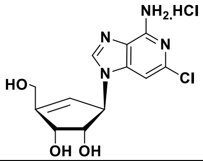
**7.**	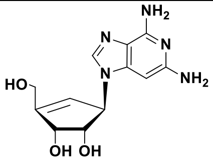
**8.**	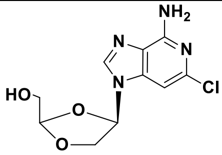
**9.**	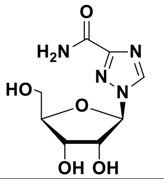

Supplementary Figure 1 – Chemical structures of compounds used in Figure 1.

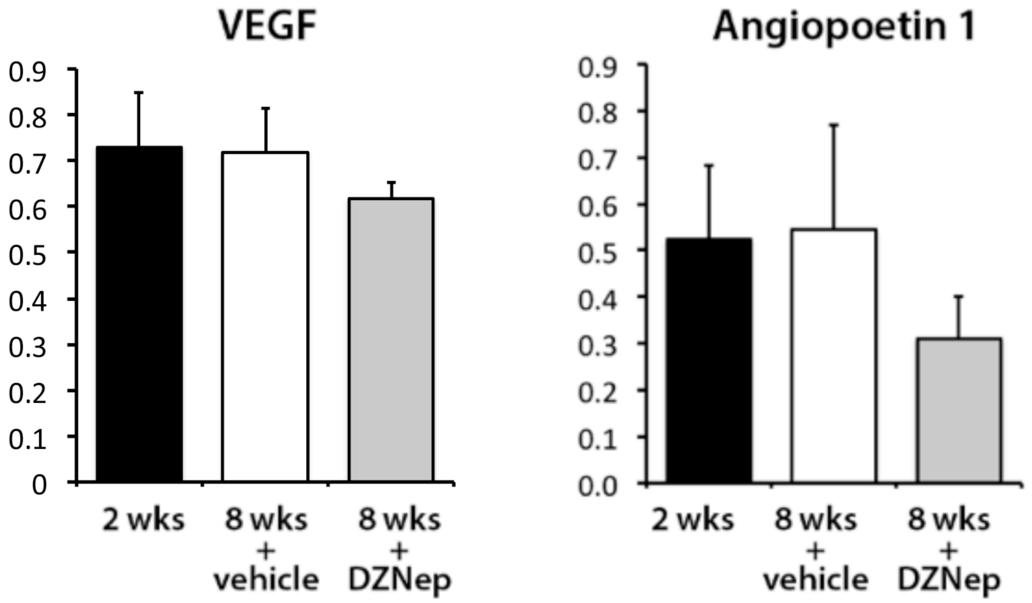

<u>Supplementary Figure 2</u> –Angiopoietin I and VEGF expression in livers of mice treated with vehicle or DZNep in chronic CCl_4 model of liver fibrosis.

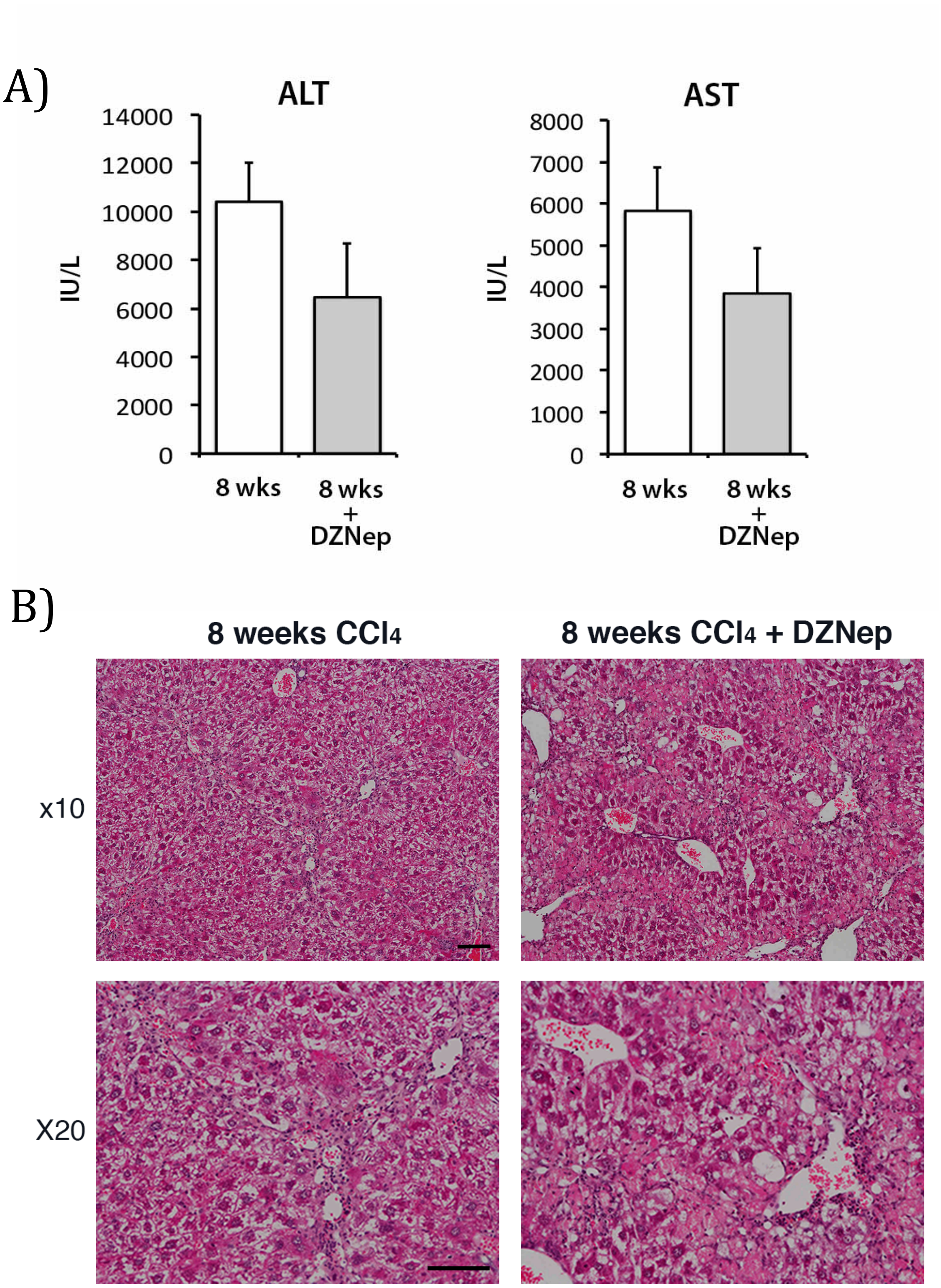

Supplementary Figure 3 – A) ALT and AST values for mice treated with DZNep in chronic CCl_4 model of liver fibrosis. B) Representative Hematoxylin and Eosin stained slides of 8 week CCl4 injured livers, with or without DZNep (or vehicle)

in bottom panels. Amplification is 10X in top two pictures and 20X in the bottom ones. Scale bar – 100mm.

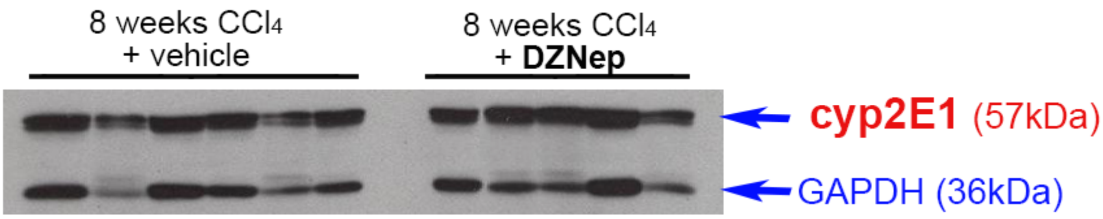

Supplementary Figure 4- Western blot for cyp2E1 expression in livers of animals receiving chronic CCl_4 with vehicle or DZNep treatment

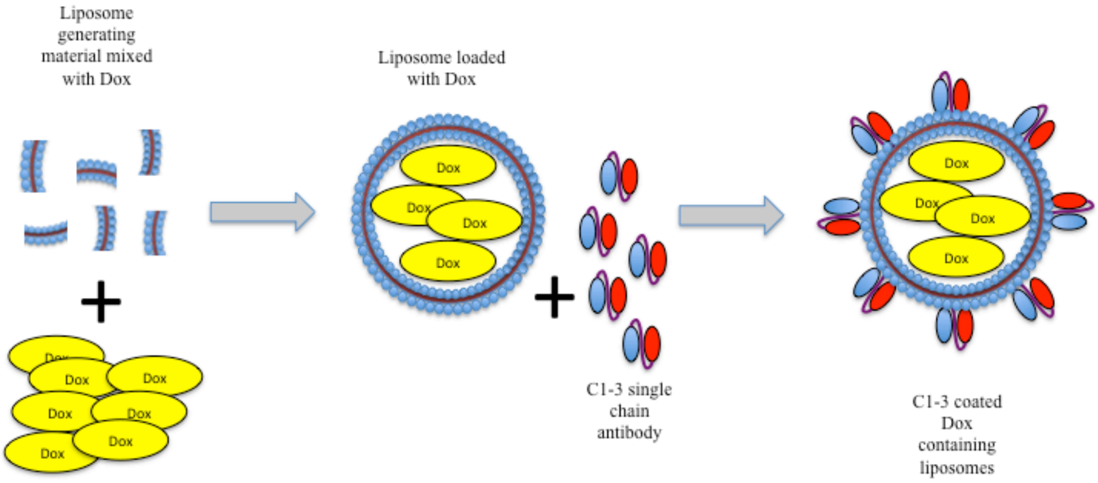

Supplementary Figure 5- Schematic of the C1-3 coated doxorubicin containing liposomes

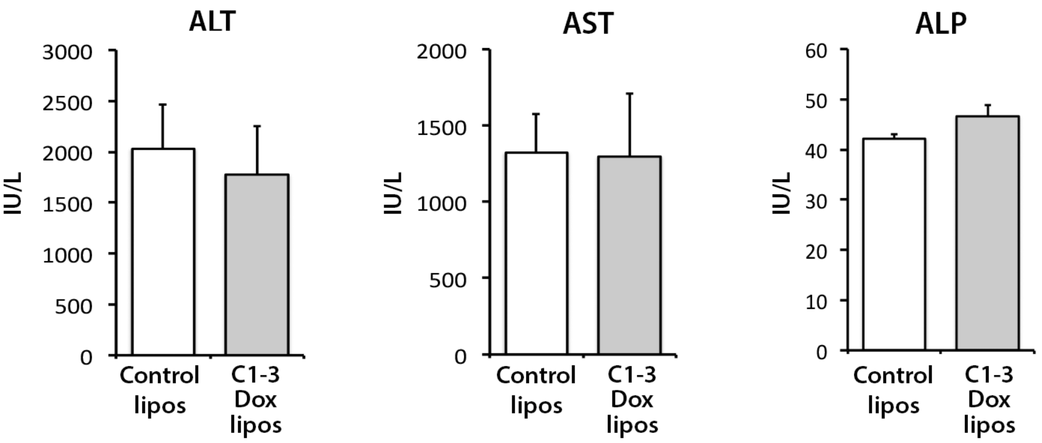

Supplementary Figure 6- ALT, AST and ALP values for mice treated with C1-3/empty liposomes (control liposomes) or C1-3/doxorubicine containing liposomes in acute CCl_4 liver injury

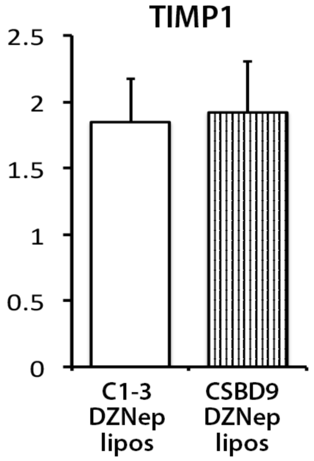

Supplementary Figure 7- TIMP1 expression in livers of mice treated with C1-3/DZNep liposomes or CSBD9/DZNep liposomes in chronic CCl_4 model of liver fibrosis

Table 2- Genes upregulated in DZNep treated livers (Sorted via fold change)

Gene name	Fold change
G6pc, glucose-6-phosphatase, catalytic, 3370255	4.4897
Hamp2, hepcidin antimicrobial peptide 2, 7330482	3.9385
Cyp4a14, cytochrome P450, family 4, subfamily a, polypeptide 14, 1940273	3.3069
Thrsp, thyroid hormone responsive SPOT14 homolog (Rattus), 6580403	3.2759
Cyp8b1, cytochrome P450, family 8, subfamily b, polypeptide 1, 4900341	3.1924
Cyp7a1, cytochrome P450, family 7, subfamily a, polypeptide 1, 4880333	3.1865
Cyp2c37, cytochrome P450, family 2. subfamily c, polypeptide 37, 4480437	3.0023
Cyp2c50, cytochrome P450, family 2, subfamily c, polypeptide 50, 290437	2.9735
Cyp2c37, cytochrome P450, family 2. subfamily c, polypeptide 37, 1240592	2.9628
Car3, carbonic anhydrase 3, 1450242	2.8145
2810007J24Rik, RIKEN cDNA 2810007J24 gene, 7610520	2.738
Acss2, acyl-CoA synthetase short-chain family member 2, 4570333	2.6234
Inmt, indolethylamine N-methyltransferase, 2360050	2.6051
Bhmt, betaine-homocysteine methyltransferase, 2480039	2.5697
Upp2, uridine phosphorylase 2, 2070372	2.53
Aqp8, aquaporin 8, 2470717	2.4786
Serpina6, serine (or cysteine) peptidase inhibitor, clade A, member 6, 6660403	2.4424
Cyp2c29, cytochrome P450, family 2, subfamily c, polypeptide 29, 4730403	2.3787
Upp2, uridine phosphorylase 2, 3520382	2.364
Slc25a25, solute carrier family 25 (mitochondrial carrier, phosphate carrier), member 25, 3440070	2.2409
Chrna4, cholinergic receptor, nicotinic, alpha polypeptide 4, 2260082	2.2151
Aqp8, aquaporin 8, 7160093	2.1861
Uhrf1, ubiquitin-like, containing PHD and RING finger domains, 1, 4560397	2.1823
Upp2, uridine phosphorylase 2, 1410170	2.0986
Raet1b, retinoic acid early transcript beta, 50025	2.0245
Cyp1a2, cytochrome P450, family 1, subfamily a, polypeptide 2, 5260367	1.9878
Slc2a2, solute carrier family 2 (facilitated glucose transporter), member 2, 2680593	1.9873
Cyp1a2, cytochrome P450, family 1, subfamily a, polypeptide 2, 1050079	1.9858
Raet1b, retinoic acid early transcript beta, 10541	1.9722
Gstm2, glutathione S-transferase, mu 2, 2690025	1.9349
Chrna4, cholinergic receptor, nicotinic, alpha polypeptide 4, 2070482	1.9326

Cyp4a31, cytochrome P450, family 4, subfamily a, polypeptide 31, 60364	1.8999
Gstm2, glutathione S-transferase, mu 2, 7510072	1.8703
Elovl6, ELOVL family member 6, elongation of long chain fatty acids (yeast), 670608	1.8645
Cyp4f14, cytochrome P450, family 4, subfamily f, polypeptide 14, 4640041	1.8626
Igfbp2, insulin-like growth factor binding protein 2, 580364	1.861
Aacs, acetoacetyl-CoA synthetase, 7650468	1.8587
Mcm6, minichromosome maintenance deficient 6 (MIS5 homolog, S. pombe) (S. cerevisiae), 3990243	1.8521
Upp2, uridine phosphorylase 2, 6040400	1.803
Mcm5, minichromosome maintenance deficient 5, cell division cycle 46 (S. cerevisiae), 6220270	1.7868
Rnf125, ring finger protein 125, 2320176	1.7865
Aldh1a1, aldehyde dehydrogenase family 1, subfamily A1, 5810470	1.7734
Gstm2, glutathione S-transferase, mu 2, 730025	1.7644
Gstt3, glutathione S-transferase, theta 3, 2350324	1.7562
Hpd, 4-hydroxyphenylpyruvic acid dioxygenase, 2360528	1.749
Pygl, liver glycogen phosphorylase, 1030142	1.7436
Agxt, alanine-glyoxylate aminotransferase, 6180408	1.7402
Psma3, proteasome (prosome, macropain) subunit, alpha type 3; 3' UTR, 5900047	1.7292
Rrm2, ribonucleotide reductase M2, 3440725	1.7222
Lss, lanosterol synthase, 5890553	1.7121
Sucnr1, succinate receptor 1, 1190148	1.7105
Mcm6, minichromosome maintenance deficient 6 (MIS5 homolog, S. pombe) (S. cerevisiae), 3290437	1.7096
Khk, ketohexokinase, 20010	1.7084
Khk, ketohexokinase, 6840014	1.7051
Psen2, presenilin 2, 990682	1.7015
Acly, ATP citrate lyase, 7050274	1.6998
Igfbp2, insulin-like growth factor binding protein 2, 1230240	1.6886
Fdps, farnesyl diphosphate synthetase, 5290671	1.6818
Dmgdh, dimethylglycine dehydrogenase precursor, 2450750	1.6815
Cyp2c70, cytochrome P450, family 2, subfamily c, polypeptide 70, 4280722	1.6791
Pah, phenylalanine hydroxylase, 1980328	1.6775
Ttc39c, tetratricopeptide repeat domain 39C, 7330228	1.6676
Slc47a1, solute carrier family 47, member 1, 6520022	1.6615
Scd1, stearoyl-Coenzyme A desaturase 1, 3890274	1.6552
Paqr9, progestin and adipoQ receptor family member IX, 650731	1.6489
Ces2g, carboxylesterase 2G, 7100458	1.6421
LOC100040592, PREDICTED: Mus musculus similar to Hmgcs1 protein, transcript variant 1 (LOC100040592), mRNA., 6940521	1.641

Paox, polyamine oxidase (exo-N4-amino), 3140102	1.6408
Insig1, insulin induced gene 1, 10309	1.6386
Gstt1, glutathione S-transferase, theta 1, 430564	1.6366
Dhcr7, 7-dehydrocholesterol reductase, 4280112	1.633
Nudt7, nudix (nucleoside diphosphate linked moiety X)-type motif 7, 5420685	1.6288
Tk1, thymidine kinase 1, 7400142	1.6261
Cyp2c67, cytochrome P450, family 2, subfamily c, polypeptide 67, 1170600	1.6232
Aldh1l1, aldehyde dehydrogenase 1 family, member L1, 380754	1.6209
Dhcr24, 24-dehydrocholesterol reductase, 4250228	1.6131
Idi1, Mus musculus isopentenyl-diphosphate delta isomerase (Idi1), transcript variant 2, mRNA., 3140768	1.6109
Slco1b2, solute carrier organic anion transporter family, member 1b2, 4570053	1.6097
Dak, dihydroxyacetone kinase 2 homolog (yeast), 1660739	1.6067
Bbox1, butyrobetaine (gamma), 2-oxoglutarate dioxygenase 1 (gamma-butyrobetaine hydroxylase), 450072	1.604
Slc22a1, solute carrier family 22 (organic cation transporter), member 1, 4250100	1.6039
Hsd17b6, hydroxysteroid (17-beta) dehydrogenase 6, 4060364	1.6017
LOC100044204, PREDICTED: Mus musculus hypothetical protein LOC100044204 (LOC100044204), mRNA., 3830048	1.5969
Mcm6, minichromosome maintenance deficient 6 (MIS5 homolog, S. pombe) (S. cerevisiae), 270379	1.5969
Pmvk, phosphomevalonate kinase, 670025	1.5963
Apol9b, apolipoprotein L 9b, 4280093	1.5951
Ugt1a6a, UDP glucuronosyltransferase 1 family, polypeptide A6A, 1170349	1.5777
Rdh11, retinol dehydrogenase 11, 650411	1.5716
Gulo, gulonolactone (L-) oxidase, 6650674	1.571
Idh2, isocitrate dehydrogenase 2 (NADP+), mitochondrial, 2510390	1.559
Hist1h2ad, histone cluster 1, H2ad, 3520717	1.5586
Hist1h2ap, histone cluster 1, H2ap, 6510253	1.5585
Ndrg2, N-myc downstream regulated gene 2, 1450601	1.5584
Adh4, alcohol dehydrogenase 4 (class II), pi polypeptide, 6840193	1.5574
Pygl, liver glycogen phosphorylase, 3310333	1.5572
Gsta3, glutathione S-transferase, alpha 3, 5550075	1.5566
Ddt, D-dopachrome tautomerase, 1990731	1.5565
Kynu, kynureninase (L-kynurenine hydrolase), 520138	1.5528
Cml1, camello-like 1, 7380671	1.5504
Mcm4, minichromosome maintenance deficient 4 homolog (S. cerevisiae), 2320368	1.5482
Abcb11, ATP-binding cassette, sub-family B (MDR/TAP), member 11,	1.5433

6330731	
Ces1g, carboxylesterase 1G, 6480397	1.5428
Dhcr24, NA, 2100162	1.5404
Hist1h2an, histone cluster 1, H2an, 4610129	1.5393
Acat2, acetyl-Coenzyme A acetyltransferase 2, 110661	1.5386
LOC100047200, PREDICTED: Mus musculus similar to T-box 3 protein (LOC100047200), mRNA., 6510162	1.538
Cyp3a25, cytochrome P450, family 3, subfamily a, polypeptide 25, 7570017	1.5379
Fignl1, fidgetin-like 1, 670500	1.5364
Dhdh, dihydrodiol dehydrogenase (dimeric), 3060066	1.536
Aox3, aldehyde oxidase 3, 3190646	1.5337
Cmbl, carboxymethylenebutenolidase-like (Pseudomonas), 5910072	1.5336
Cyp2b9, cytochrome P450, family 2, subfamily b, polypeptide 9, 1940504	1.5287
Ly6d, lymphocyte antigen 6 complex, locus D, 2510646	1.5262
Paqr9, progestin and adipoQ receptor family member IX, 3440739	1.5238
Hist1h2af, histone cluster 1, H2af, 4250711	1.5222
Tcf19, transcription factor 19, 6330725	1.5213
Nat8, N-acetyltransferase 8 (GCN5-related, putative), 3170255	1.5207
E2f1, E2F transcription factor 1, 60369	1.5139
Cisd1, CDGSH iron sulfur domain 1, 110576	1.513
Hsd3b7, hydroxy-delta-5-steroid dehydrogenase, 3 beta- and steroid delta-isomerase 7, 5890494	1.5122
Slc2a2, solute carrier family 2 (facilitated glucose transporter), member 2, 630487	1.5117
Ddc, dopa decarboxylase, 610601	1.5084
Slc2a2, solute carrier family 2 (facilitated glucose transporter), member 2, 2760463	1.5065
Ly6d, lymphocyte antigen 6 complex, locus D, 5560754	1.5031
Aldh1a7, aldehyde dehydrogenase family 1, subfamily A7, 3130288	1.5019
Sc4mol, sterol-C4-methyl oxidase-like, 6420253	1.5002
Hist1h2ak, histone cluster 1, H2ak, 3130609	1.4995
Cyp51, cytochrome P450, family 51, 540020	1.4962
Sc5d, sterol-C5-desaturase (fungal ERG3, delta-5-desaturase) homolog (S. cerevisae), 4150747	1.4952
Pecr, peroxisomal trans-2-enoyl-CoA reductase, 2260561	1.4937
Rarres2, retinoic acid receptor responder (tazarotene induced) 2, 6060367	1.4926
Cyp2e1, cytochrome P450, family 2, subfamily e, polypeptide 1, 6280133	1.4923
Rgn, regucalcin, 3440437	1.4866
LOC100047937, PREDICTED: Mus musculus similar to Aldehyde dehydrogenase 1 family, member L1 (LOC100047937), mRNA., 4890608	1.4863
Rnf125, ring finger protein 125, 4050541	1.4862
Ces1d, carboxylesterase 1D, 670603	1.481

Gamt, guanidinoacetate methyltransferase, 290044	1.4792
Mthfd1, methylenetetrahydrofolate dehydrogenase (NADP+ dependent), methenyltetrahydrofolate cyclohydrolase, formyltetrahydrofolate synthase, 7150484	1.4787
Afm, afamin, 5890706	1.4784
Ttc39c, tetratricopeptide repeat domain 39C, 4670022	1.4757
Rfc4, replication factor C (activator 1) 4, 3940458	1.4749
Tlcd2, TLC domain containing 2, 5900592	1.4718
Apoa5, apolipoprotein A-V, 7380762	1.4717
Cdt1, chromatin licensing and DNA replication factor 1, 1050706	1.4713
Insig1, insulin induced gene 1, 5310343	1.4662
Dcxr, dicarbonyl L-xylulose reductase, 3140274	1.4643
1100001G20Rik, RIKEN cDNA 1100001G20 gene, 1300707	1.4623
Cyp2c55, cytochrome P450, family 2, subfamily c, polypeptide 55, 2360427	1.4618
Fen1, flap structure specific endonuclease 1, 3460037	1.4616
Ephx2, epoxide hydrolase 2, cytoplasmic, 3520491	1.4603
Cyp2f2, cytochrome P450, family 2, subfamily f, polypeptide 2, 6040689	1.4596
Ebpl, emopamil binding protein-like, 110630	1.4572
Sult1a1, sulfotransferase family 1A, phenol-preferring, member 1, 4070215	1.4568
Tecr, trans-2,3-enoyl-CoA reductase, 60220	1.4561
Tecr, trans-2,3-enoyl-CoA reductase, 6620603	1.4551
D0H4S114, DNA segment, human D4S114, 7040243	1.4548
Cdc6, cell division cycle 6 homolog (S. cerevisiae), 2030026	1.4542
Tmie, transmembrane inner ear, 5390632	1.4522
4931406C07Rik, RIKEN cDNA 4931406C07 gene, 630576	1.4501
Lig1, ligase I, DNA, ATP-dependent, 3060767	1.4481
Abat, 4-aminobutyrate aminotransferase, 6020181	1.4472
Haao, 3-hydroxyanthranilate 3,4-dioxygenase, 1740164	1.4452
LOC668837, PREDICTED: Mus musculus similar to ATP synthase, H+ transporting, mitochondrial F0 complex, subunit G (LOC668837), misc RNA., 2690097	1.4445
Paox, polyamine oxidase (exo-N4-amino), 4120470	1.4445
Sec14l2, SEC14-like 2 (S. cerevisiae), 1260075	1.4416
Spp2, secreted phosphoprotein 2, 4860068	1.438
Mrap, melanocortin 2 receptor accessory protein, 5870487	1.4328
Olfml1, olfactomedin-like 1, 4780020	1.4327
Nrn1, neuritin 1, 5050471	1.4321
Gstm4, glutathione S-transferase, mu 4, 2320228	1.4312
Pank1, pantothenate kinase 1, 6290411	1.4311
Spc24, SPC24, NDC80 kinetochore complex component, homolog (S. cerevisiae), 5340398	1.4245
Rrm2, ribonucleotide reductase M2, 5560646	1.4228

Apon, apolipoprotein N, 7400376	1.421
Tmem86b, transmembrane protein 86B, 6250184	1.4204
Hist1h2ah, histone cluster 1, H2ah, 1470341	1.4176
Tdo2, tryptophan 2,3-dioxygenase, 4180187	1.4163
Tecr, trans-2,3-enoyl-CoA reductase, 5900333	1.4162
Pon1, paraoxonase 1, 4390398	1.4152
Baat, bile acid-Coenzyme A: amino acid N-acyltransferase, 2510674	1.4148
Nsdhl, NAD(P) dependent steroid dehydrogenase-like, 2650653	1.4139
Vkorc1, vitamin K epoxide reductase complex, subunit 1, 7650435	1.4119
Selenbp2, selenium binding protein 2, 4920725	1.4108
Slco1b2, solute carrier organic anion transporter family, member 1b2, 1410035	1.4094
Rpa1, replication protein A1, 1340671	1.4085
Atp5a1, ATP synthase, H+ transporting, mitochondrial F1 complex, alpha subunit 1, 6020746	1.4073
Dhcr24, 24-dehydrocholesterol reductase, 6370681	1.4071
Ebpl, emopamil binding protein-like, 5810722	1.4071
Ccl9, chemokine (C-C motif) ligand 9, 7050538	1.4043
Sord, sorbitol dehydrogenase, 5720014	1.4043
Fam73b, family with sequence similarity 73, member B, 1170537	1.4037
Aldh1b1, aldehyde dehydrogenase 1 family, member B1, 2650154	1.4029
Bhmt2, betaine-homocysteine methyltransferase 2, 610576	1.401
Dhdh, dihydrodiol dehydrogenase (dimeric), 830195	1.4003
Hsd17b7, hydroxysteroid (17-beta) dehydrogenase 7, 1070097	1.4001
Fahd1, fumarylacetoacetate hydrolase domain containing 1, 4120692	1.3999
Sgk2, serum/glucocorticoid regulated kinase 2, 3520519	1.3999
Gchfr, GTP cyclohydrolase I feedback regulator, 1340711	1.3989
Slc38a3, solute carrier family 38, member 3, 6100154	1.3958
Reln, reelin, 7610484	1.3955
Tcea3, transcription elongation factor A (SII), 3, 2650372	1.395
Ebp, phenylalkylamine Ca2+ antagonist (emopamil) binding protein, 1990112	1.3942
Cyp27a1, cytochrome P450, family 27, subfamily a, polypeptide 1, 2940170	1.393
Sephs2, selenophosphate synthetase 2, 1770707	1.3923
Rbp4, retinol binding protein 4, plasma, 5420240	1.3914
5730469M10Rik, RIKEN cDNA 5730469M10 gene, 3180386	1.3905
0610007P14Rik, RIKEN cDNA 0610007P14 gene, 1230730	1.3881
Klk1b4, kallikrein 1-related pepidase b4, 1090333	1.3867
Slc25a44, solute carrier family 25, member 44, 5550243	1.3866
Hpgd, hydroxyprostaglandin dehydrogenase 15 (NAD), 2450343	1.3863
Slc38a4, solute carrier family 38, member 4, 5340386	1.386
Lpcat3, lysophosphatidylcholine acyltransferase 3, 2680703	1.384

Ddah1, dimethylarginine dimethylaminohydrolase 1, 2060592	1.3834
Akr1c14, aldo-keto reductase family 1, member C14, 1690632	1.3833
Nfic, nuclear factor I/C, 3840059	1.3818
Mvd, mevalonate (diphospho) decarboxylase, 2100097	1.3818
Entpd5, ectonucleoside triphosphate diphosphohydrolase 5, 2320292	1.3803
Cyp3a11, cytochrome P450, family 3, subfamily a, polypeptide 11, 7320431	1.3801
Cyb5r3, cytochrome b5 reductase 3, 840309	1.3786
Amy1, amylase 1, salivary, 4850164	1.3775
Cyp17a1, cytochrome P450, family 17, subfamily a, polypeptide 1, 670653	1.3773
Hacl1, 2-hydroxyacyl-CoA lyase 1, 7210615	1.3765
Acot3, acyl-CoA thioesterase 3, 2230600	1.3761
Nit2, nitrilase family, member 2, 1090136	1.3759
Ugt2a3, UDP glucuronosyltransferase 2 family, polypeptide A3, 3710040	1.3757
Gstk1, glutathione S-transferase kappa 1, 5890487	1.3709
Mgst3, microsomal glutathione S-transferase 3, 4210619	1.3701
Ces1e, carboxylesterase 1E, 4040259	1.3686
Ttpa, tocopherol (alpha) transfer protein, 7000431	1.3652
Hyi, hydroxypyruvate isomerase homolog (E. coli), 4830576	1.3651
Nsdhl, NAD(P) dependent steroid dehydrogenase-like, 4830717	1.3642
Nsdhl, NAD(P) dependent steroid dehydrogenase-like, 990338	1.3569
Cat, catalase, 4760356	1.3537
Gsta3, glutathione S-transferase, alpha 3, 3780193	1.352
Otc, ornithine transcarbamylase, 4890731	1.3499
Cox7b, cytochrome c oxidase subunit VIIb, 940692	1.3489
Gpld1, glycosylphosphatidylinositol specific phospholipase D1, 6940064	1.3474
Nsdhl, NAD(P) dependent steroid dehydrogenase-like, 5820603	1.3469
Serpinc1, serine (or cysteine) peptidase inhibitor, clade C (antithrombin), member 1, 1690128	1.3458
Pcyt1a, phosphate cytidylyltransferase 1, choline, alpha isoform, 150475	1.3454
Acsm3, acyl-CoA synthetase medium-chain family member 3, 3170270	1.3451
Stard4, StAR-related lipid transfer (START) domain containing 4, 4210288	1.344
Snurf, SNRPN upstream reading frame, 3130246	1.3427
Insig1, insulin induced gene 1, 4920369	1.3405
Pemt, phosphatidylethanolamine N-methyltransferase, 620349	1.3257
Ces2e, carboxylesterase 2E, 780333	1.3239
Pmpcb, peptidase (mitochondrial processing) beta, 450544	1.3196
Hsd11b1, hydroxysteroid 11-beta dehydrogenase 1, 2340301	1.3103

Table 3- Genes downregulated in DZNep treated livers (Sorted via fold change)

Gene name	Fold change
Hbb-b1, hemoglobin, beta adult major chain, 670403	0.4458
Slpi, secretory leukocyte peptidase inhibitor, 2810487	0.4501
Trib3, tribbles homolog 3 (Drosophila), 4280056	0.4549
Hba-a1, hemoglobin alpha, adult chain 1, 2000398	0.4659
Mup21, major urinary protein 21, 3440110	0.4864
Egr1, early growth response 1, 6620079	0.4913
Chac1, ChaC, cation transport regulator-like 1 (E. coli), 540300	0.4955
Ctgf, connective tissue growth factor, 4010082	0.503
Acta2, actin, alpha 2, smooth muscle, aorta, 430068	0.5044
Creld2, cysteine-rich with EGF-like domains 2, 4860079	0.519
Tnfrsf12a, tumor necrosis factor receptor superfamily, member 12a, 1770541	0.5239
Gadd45a, growth arrest and DNA-damage-inducible 45 alpha, 580609	0.536
Gadd45a, growth arrest and DNA-damage-inducible 45 alpha, 3890332	0.5424
Creld2, cysteine-rich with EGF-like domains 2, 290685	0.545
Hspb1, heat shock protein 1, 5670722	0.5471
Emp1, epithelial membrane protein 1, 7160167	0.5534
Hspa8, heat shock protein 8, 2030593	0.571
Ddit3, DNA-damage inducible transcript 3, 1500497	0.573
Syvn1, synovial apoptosis inhibitor 1, synoviolin, 10674	0.5743
Xlr4a, X-linked lymphocyte-regulated 4A, 5870021	0.5867
Herpud1, homocysteine-inducible, endoplasmic reticulum stress-inducible, ubiquitin-like domain member 1, 1050619	0.5935
Herpud1, homocysteine-inducible, endoplasmic reticulum stress-inducible, ubiquitin-like domain member 1, 50129	0.5947
Gtpbp2, GTP binding protein 2, 3850142	0.6031
Cxcl9, chemokine (C-X-C motif) ligand 9, 10598	0.6049
Npy, neuropeptide Y, 160494	0.6069
Plin2, perilipin 2, 5670164	0.6082
Dnajc12, DnaJ (Hsp40) homolog, subfamily C, member 12, 1230441	0.6091
Acta2, actin, alpha 2, smooth muscle, aorta, 2140255	0.6118
Pdlim7, PDZ and LIM domain 7, 6550609	0.6165
Acta2, actin, alpha 2, smooth muscle, aorta, 1850022	0.6281
Cyp2a5, cytochrome P450, family 2, subfamily a, polypeptide 5, 840114	0.633
Oat, ornithine aminotransferase, 4560309	0.6417
Osgin1, oxidative stress induced growth inhibitor 1, 430037	0.6426
Acot1, acyl-CoA thioesterase 1, 450356	0.6447

Tes, testis derived transcript, 7550121	0.6448
Saa3, serum amyloid A 3, 6400719	0.6456
Cd14, CD14 antigen, 6020674	0.6486
Cxcl1, chemokine (C-X-C motif) ligand 1, 3610082	0.6494
Sprr1b, small proline-rich protein 1B, 6450228	0.6503
Zfand2a, zinc finger, AN1-type domain 2A, 1400170	0.6525
Tfrc, transferrin receptor, 620110	0.6552
Slc38a2, solute carrier family 38, member 2, 4640008	0.6559
Ch25h, cholesterol 25-hydroxylase, 1510349	0.6583
S100a8, S100 calcium binding protein A8 (calgranulin A), 1190546	0.661
Cnot3, CCR4-NOT transcription complex, subunit 3; exon 12, 130519	0.6617
Cd9, CD9 antigen, 3990674	0.6626
Ms4a6d, membrane-spanning 4-domains, subfamily A, member 6D, 3180025	0.6634
Ctsj, cathepsin J, 1170041	0.6639
Mt1, metallothionein 1, 5220279	0.6644
Nupr1, nuclear protein 1, 1240424	0.6655
Lgmn, legumain, 1690719	0.6699
Cyp2a5, cytochrome P450, family 2, subfamily a, polypeptide 5, 6350333	0.6701
Hsph1, heat shock 105kDa/110kDa protein 1, 3290270	0.6703
Rhbdf1, rhomboid family 1 (Drosophila), 3840598	0.6715
Tfrc, transferrin receptor, 1780524	0.6718
Foxq1, forkhead box Q1, 1850487	0.6731
Tmsb10, thymosin, beta 10, 5130273	0.6738
P2rx4, purinergic receptor P2X, ligand-gated ion channel 4, 2030484	0.6746
Acta2, actin, alpha 2, smooth muscle, aorta, 3130136	0.677
Arpc1b, actin related protein 2/3 complex, subunit 1B, 3120576	0.6783
Cd63, CD63 antigen, 7050487	0.6799
1500012F01Rik, RIKEN cDNA 1500012F01 gene, 540138	0.6808
Cyp2a5, cytochrome P450, family 2, subfamily a, polypeptide 5, 1500180	0.6821
Tbrg1, transforming growth factor beta regulated gene 1, 1450220	0.6831
Eef2, eukaryotic translation elongation factor 2, 1690070	0.685
Sqstm1, sequestosome 1, 4560619	0.6889
Apcs, serum amyloid P-component, 2320170	0.6895
Lgals3, lectin, galactose binding, soluble 3, 510474	0.6919
Nfkbiz, nuclear factor of kappa light polypeptide gene enhancer in B cells inhibitor, zeta, 6650458	0.692
Mtap7d1, microtubule-associated protein 7 domain containing 1, 670543	0.6975
Ddit3, DNA-damage inducible transcript 3, 1230605	0.6993
Chordc1, cysteine and histidine-rich domain (CHORD)-containing, zinc-binding protein 1, 360725	0.7003
Hist1h1c, histone cluster 1, H1c, 50079	0.701
Fgf21, fibroblast growth factor 21, 6290743	0.7011

Uap1l1, UDP-N-acteylglucosamine pyrophosphorylase 1-like 1, 1410113	0.7022
Zyx, zyxin, 4050400	0.7041
S100a9, S100 calcium binding protein A9 (calgranulin B), 1980603	0.705
S100a6, S100 calcium binding protein A6 (calcyclin), 5080326	0.7052
Ddx17, DEAD (Asp-Glu-Ala-Asp) box polypeptide 17, 6180465	0.7066
Wfdc3, WAP four-disulfide core domain 3, 1570370	0.7067
Srxn1, sulfiredoxin 1 homolog (S. cerevisiae), 2970189	0.707
P2rx4, purinergic receptor P2X, ligand-gated ion channel 4, 3120341	0.7101
Lims2, LIM and senescent cell antigen like domains 2, 3180681	0.7107
Ugt1a6a, UDP glucuronosyltransferase 1 family, polypeptide A6A, 3800446	0.7114
Cd63, CD63 antigen, 5090053	0.7119
Col23a1, procollagen, type 23, alpha 1; 3' UTR, 2030358	0.7133
Dnajb1, DnaJ (Hsp40) homolog, subfamily B, member 1, 4540020	0.7161
Foxa3, forkhead box A3, 5290376	0.7162
Dnttip1, deoxynucleotidyltransferase, terminal, interacting protein 1, 4920072	0.7166
Fndc3b, fibronectin type III domain containing 3B, 7160364	0.7176
Vmp1, vacuole membrane protein 1, 5690327	0.7178
Egfr, epidermal growth factor receptor, 1770292	0.719
Egfr, epidermal growth factor receptor, 4540382	0.7194
Tnfrsf22, tumor necrosis factor receptor superfamily, member 22, 430301	0.7195
Gm11428, predicted gene 11428, 5360370	0.7218
Nrbp2, nuclear receptor binding protein 2, 110561	0.7258
Bag3, BCL2-associated athanogene 3, 6660653	0.7262
Psat1, phosphoserine aminotransferase 1, 2260010	0.727
Tmem86a, transmembrane protein 86A, 2750184	0.7273
Hspa5, heat shock protein 5, 150678	0.7277
Atp2a2, ATPase, Ca++ transporting, cardiac muscle, slow twitch 2, 770349	0.731
Rbpms, RNA binding protein gene with multiple splicing, 4050121	0.7318
Rpl23, ribosomal protein L23, 670176	0.7396
Tmc7, transmembrane channel-like gene family 7, 5910408	0.7398
Stbd1, starch binding domain 1, 1430129	0.7403
Timp1, tissue inhibitor of metalloproteinase 1, 4640215	0.7429
Scara5, scavenger receptor class A, member 5 (putative), 160377	0.747
Dbp, D site albumin promoter binding protein, 3180750	0.796
